# Evident lower blood levels of multiple nutritional compounds and highly prevalent malnutrition in sub-acute stroke patients with or without dysphagia

**DOI:** 10.3389/fneur.2022.1028991

**Published:** 2023-01-10

**Authors:** Nick van Wijk, Bettina Studer, Claudia A. van den Berg, Dina Ripken, Mirian Lansink, Mario Siebler, Tobias Schmidt-Wilcke

**Affiliations:** ^1^Danone Nutricia Research, Utrecht, Netherlands; ^2^St. Mauritius Therapieklinik, Meerbusch, Germany; ^3^Medical Faculty, Institute for Clinical Neuroscience, University of Düsseldorf, Düsseldorf, Germany; ^4^Neurologie und Neurorehabilitation, Fachklinik Rhein Ruhr, Essen, Germany; ^5^Department of Neurology, University of Düsseldorf, Düsseldorf, Germany; ^6^Neurologisches Zentrum, Mainkofen, Germany

**Keywords:** ischemic stroke, oropharyngeal dysphagia, rehabilitation, malnutrition (MeSH), nutritional status (source: MeSH NLM), blood levels, vitamins, nutritional intake

## Abstract

**Introduction:**

Malnutrition is prevalent after stroke, particularly if post-stroke oropharyngeal dysphagia (OD) reduces nutritional intake. To further understand stroke-related malnutrition, a thorough nutritional assessment was performed in ischemic stroke patients with or without OD during sub-acute inpatient rehabilitation.

**Methods:**

In this exploratory, observational, cross-sectional, multi-center study in Germany (NTR6802), ischemic stroke patients with (*N* = 36) or without (*N* = 49) OD were age- and sex-matched to healthy reference subjects. Presence of (risk of) malnutrition (MNA-SF), blood concentration of stroke-relevant nutritional compounds and metabolites, nutritional intake, quality of life (EQ-5D-5L), and activities of daily living (Barthel index) were assessed.

**Results:**

More than half of the stroke patients displayed (risk of) malnutrition, with higher prevalence in patient with OD *vs*. without OD. Fasted blood concentrations of vitamins B1, B2, B6, A, D, and E, selenium, choline, coenzyme Q10, albumin, pre-albumin, transferrin, docosahexaenoic acid, and eicosapentaenoic acid were all lower in stroke patients compared to their matched healthy reference subjects, irrespective of OD status. Reported energy, macronutrient, and water intake were lower in stroke patients *vs*. healthy reference subjects. As expected, quality of life and activities of daily living scores were lower in stroke *vs*. healthy reference subjects, with OD scoring worse than non-OD patients.

**Discussion:**

This study shows that malnutrition is highly prevalent in sub-acute stroke patients during rehabilitation. Even though patients with OD were more likely to be malnourished, blood levels of specific nutritional compounds were similarly lower in stroke patients with or without OD compared to healthy reference subjects. Furthermore, subgroup analysis showed similarly lower blood levels of specific nutritional compounds in patients that are normal nourished *vs*. patients with (risk of) malnutrition. This might imply disease-specific changes in blood levels on top of overall protein-energy malnutrition. The results of the current study underline that it is important to screen for nutritional impairments in every stroke patient, either with or without OD.

## Introduction

Deterioration in nutritional status is highly prevalent after stroke. Notwithstanding the wide variation in reported prevalence of malnutrition amongst different studies (1), a recent meta-analysis reported a pooled prevalence of malnutrition after stroke of 19% in the hyperacute phase up to 37% in the early subacute phase and 30% chronic phase (2). Factors that expedite stroke-related malnutrition include impaired consciousness, perception deficits, cognitive dysfunction, paresis, and most notably oropharyngeal dysphagia (OD) (3, 4).

OD frequently occurs after stroke, although studies report a wide variation in prevalence because of differences in dysphagia assessments, stroke type, timing, and setting. The prevalence of dysphagia is highest in acute stroke, occurring in between one and three quarters of the patients (5). Spontaneous recovery occurs in the first weeks after stroke in many patients (6). However, prevalence of chronic OD has also been reported to be as high as 50% at 6 months (7).

While post-stroke OD increases the risk of malnutrition (8), the consequences of malnutrition may also affect patients' ability to swallow (9). Both post-stroke OD and malnutrition are consistently associated with poor clinical outcomes (10, 11), where malnutrition is an independent risk factor of mortality, and length of hospital stay at 3 (12) and 6 months post stroke (11).

Stroke complications are not limited to protein-energy malnutrition but can also encompass specific micronutrients and fatty acids insufficiencies or deficiencies [e.g., (13–15)]. Such lower blood levels might result from stroke-related changes in nutrient intake, uptake, or metabolism. Although numerous studies of different quality investigated nutritional blood levels after stroke, few of them were performed in the rehabilitation setting, i.e., the subacute phase, or specified whether patients with and/or without dysphagia were included.

To better understand the extent of stroke-related malnutrition in the rehabilitation phase, a comprehensive exploratory nutritional assessment in sub-acute ischemic stroke patients with or without OD was performed.

Blood concentration of stroke-relevant nutritional compounds and metabolites, presence of (risk of) malnutrition, nutritional intake, quality of life (QoL), and activities of daily living (ADL) were assessed once between 2 and 12 weeks after stroke and compared to age- and sex-matched healthy reference subjects.

## Subjects and methods

### Study population and design

The study population comprised ischemic stroke patients (age ≥50 and ≤ 75 years) with and without oropharyngeal dysphagia (abbreviated as S+OD and S-OD, respectively) as inpatients in a rehabilitation center ≥2 and ≤ 12 weeks after stroke occurrence. Presence of dysphagia was defined as a Bogenhausener Dysphagie-Score (BODS)-2 of ≥3 (16). BODS-2 grades the ability of oral food intake (BODS-2) on a range from 1 (normal) to 8 (exclusively enteral or parenteral nutrition). Patients on tube feeding within 2 weeks prior to planned study enrolment and/or on current prescription of vitamin injections were not enrolled. Stroke patients were 1:1 matched by sex and age (range of −3 to +3 years) with healthy reference (HR) subjects with a body mass index (BMI) of ≥20 and <30 kg/m^2^. Exclusion criteria for HR subjects included: following a special diet, having diabetes mellitus type II, or using anti-hypertensive or cholesterol- or triglyceride-lowering drugs. A complete list of inclusion and exclusion criteria is provided in the Supplementary material.

The study protocol was approved by the Ethics Review Committee Ärztekammer Nordrhein in Germany (identifier: MPR16TA07987). The study was registered at the Netherlands Trial Register (identifier: NTR6802; accessible at the International Clinical Trial Registry Platform). Study procedures were performed in accordance with the Declaration of Helsinki ethical principles for medical research involving human subjects and with the International Conference on Harmonization (ICH) guidelines for Good Clinical Practice (GCP). Written informed consent was obtained from all patients and HR subjects before their inclusion in the study.

This exploratory, observational, cross-sectional, multi-center study was carried out in two rehabilitation centers (St. Mauritius Therapieklinik, Meerbusch and Fachklinik Rhein Ruhr, Neurologie und Neurorehabilitation, Essen) and in one center recruiting healthy volunteers (CRS Clinical Research Services Mönchengladbach GmbH, Mönchengladbach) in Germany.

**Figure 1:**
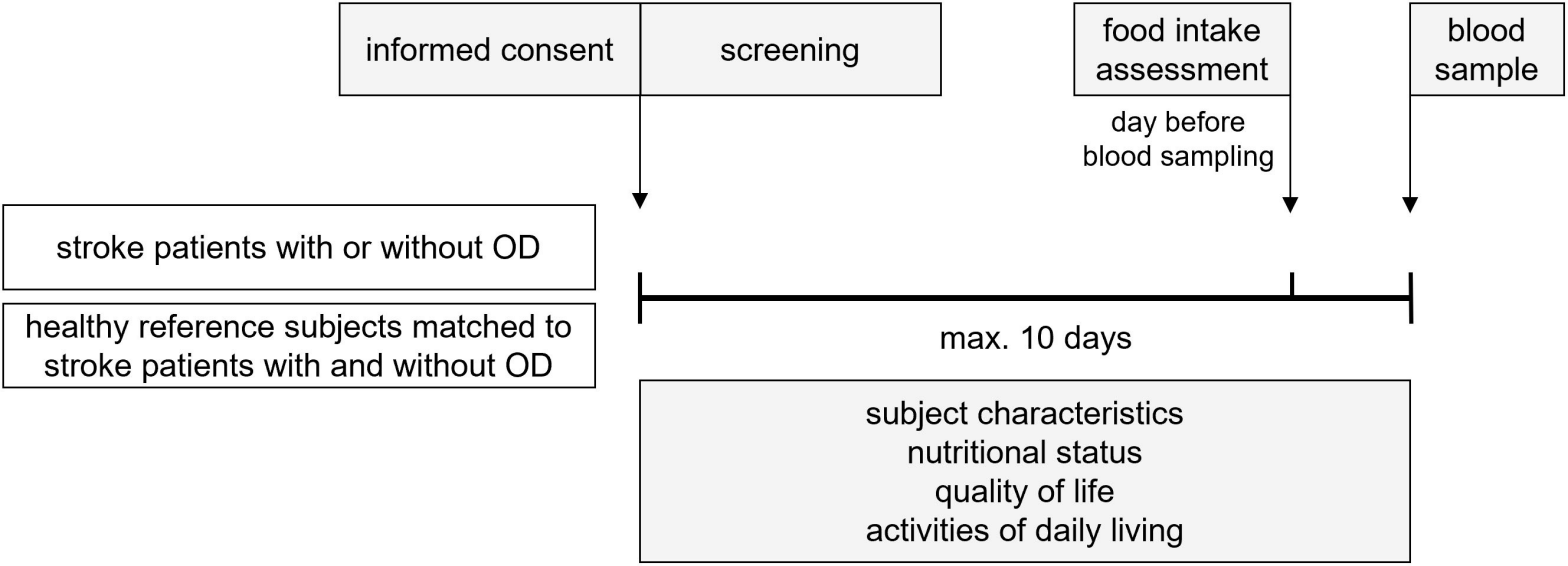
Schematic diagram of study design.

Figure 1 represents a schematic diagram of the study design. Within 10 days after screening and inclusion of eligible subjects, subject characteristics were documented and the nutritional status, quality of life, and activities of daily living were assessed. One day before blood sampling, a one day food intake was recorded. Maximal 10 days after screening and inclusion, a blood sample was collected to determine nutritional compounds and metabolites concentration. Fasted blood samples were obtained by venipuncture (50 mL in total) in the morning. To obtain a fasted state for blood sampling, subjects were asked not to eat or drink after midnight on the day before sampling, except for drinking water, which was allowed until 1 hour before the sample was taken. After collection, the blood samples were immediately processed and stored at −20 °C. Samples were sent in batches to dedicated ISO certified laboratories and stored at −80 °C till analysis.

### Outcomes

Sex, age, BMI, race, living situation (prior stroke), current marital status, socioeconomic status, educational status, smoking status, and alcohol use were documented. For the stroke patients, time since stroke, localization of stroke, National Institutes of Health Stroke Scale (NIHSS) score at hospital admittance, and occurrence of mechanical ventilation and/or tube feeding after stroke were reported. Relevant medical history, the use of concomitant medication, and medical complications during the rehabilitation phase were recorded as well. Medical history of the HR subjects was not recorded, but concomitant medication was.

Main outcome parameters were (risk of) malnutrition, nutritional compounds and metabolites concentration in blood (fasted) and nutritional intake. Malnutrition was assessed using the Mini Nutritional Assessment–Short Form (MNA-SF) questionnaire (17). This screening tool is a sum of scores [0–14] on 6-items, with the following categories: 0–7 points = malnourished; 8–11 points = at risk of malnutrition; 12–14 points = normal nourished. Fasted blood samples were obtained to analyze the following nutritional compounds and metabolites: vitamins A, B1, B2, B6, B9, B12, D, and E, folate, selenium, zinc, magnesium, choline, uridine, glucose, coenzyme Q10 (coQ10), total cholesterol, albumin, pre-albumin, transferrin, acylcarnitine and free carnitine, creatinine and creatine, C-reactive protein (CRP), sodium, and fatty acid composition. In addition, serum osmolality was analyzed. Nutritional intake was assessed with a one-day food diary. Diary data was entered in EbisPro (version 2016, Dr. J. Erhardt, Willstätt-Legelshurst, Germany), a German nutritional intake application, with which the energy, macro- and micronutrient intake were assessed. Subjects in the stroke rehabilitation group were assisted by the study staff and/or relatives to complete the food diary if needed.

Other outcome parameters were quality of life (QoL) and Activities of daily living (ADL). QoL was measured using the EuroQol 5-dimensions 5-level (EQ-5D-5L) questionnaire (18). This questionnaire gives an index value [0–1] and a visual analog scale (VAS) score [0–100]. The index value is calculated from the separate score on five dimensions: mobility, self-care, usual activities, pain/discomfort, and anxiety/depression. Each dimension has 5 levels: no problems, slight problems, moderate problems, severe problems, and extreme problems. The VAS score records the respondent's self-rated health on a 20 cm vertical, visual analog scale with endpoints labeled ‘the best health you can imagine' and ‘the worst health you can imagine' on that day. ADL was assessed with the Barthel Index questionnaire (19). The Barthel Index is a sum of scores [0–100] on ten categories to measure independent performance in activities of daily living: feeding, bathing, grooming, dressing, bowels (incontinence), bladder (incontinence), toilet use, transfers (bed to chair and back), mobility, and stairs.

Medical events, defined as any untoward medical occurrence, and their relatedness to the study procedures were recorded as safety parameter.

### Biochemical analysis

Reinier HAGA Medical Diagnostic Center (Delft, the Netherlands) analyzed the following nutritional compounds and metabolites concentration in serum: vitamin B12 and folate using Competitive Protein Binding Ligand (CPBL) with electro-chemo-luminescence; 25-hydroxyvitamin D with a chemo-luminescence micro-particle immunoassay; magnesium, creatinine, and albumin with the colorimetric method; CRP (high sensitivity measurement), transferrin, and pre-albumin with the turbidimetric method; fasting glucose and total cholesterol with enzymatic colorimetry; creatine with the chromatographic Ultra-Violet (UV) method; osmolality with assessment of freezing-point depression; and sodium with direct ion-selective electrodes. In heparin plasma, carnitine and coQ10 (ubiquinone) were analyzed with respectively Liquid Chromatography-Mass Spectrometry (LC-MS) method and with the chromatographic electrochemical method. Vitamin B1, B2, and B6 were measured with High Performance Liquid Chromatography (HPLC) and spectrofluorometry in ethylenediaminetetraacetic acid (EDTA) whole blood. Selenium and zinc were analyzed in trace element free Na-heparin plasma with Zeeman Atomic Absorption Spectrophotometry.

VUmc Clinical Chemistry Laboratory (Amsterdam, the Netherlands) analyzed free choline with LC-MS/MS in EDTA plasma.

Analytical Chemistry Laboratory Nutricia Research (Utrecht, the Netherlands) analyzed vitamin E in blood serum using Ultra-Fast Liquid Chromatograph with Fluorine detector (UFLC-Flu), and serum vitamin A and uridine using UFLC-UV detector. In citric acid plasma (specific for homocysteine detection), homocysteine was measured using UFLC-Flu method. Fatty acid composition in the total lipid fraction in EDTA plasma and erythrocytes were analyzed with gas chromatography. In contrast to plasma, erythrocytes were not quantitatively pipetted and were therefore only available in weight percentage of the total fatty acid content.

### Statistical analysis

All analyses were performed with the Per-Protocol (PP) subject data set. No sample size calculation was performed because of the exploratory nature of the study. Hundred stroke patients (50 S+OD and 50 S-OD) and 100 HR subjects (50 matched to S+OD and 50 matched to S-OD) were planned to be included.

Descriptive statistics were reported as mean (SD), and/or median (interquartile range, [IQR]) for skewed distributed data, or as *n* (%). Results were presented for stroke patients without OD (S-OD) and with OD (S+OD), and for the total stroke group (Total S) as well as for HR subjects age- and sex matched to S-OD and matched to S+OD, and for the total HR group (Total HR).

Inferential statistics were performed for the three paired comparisons: (1) S-OD *vs*. HR matched to S-OD; (2) S+OD *vs*. HR matched to S+OD; (3) Total S *vs*. Total HR; and one unpaired comparison: (4) S-OD *vs*. S+OD. For all outcome parameters, a *p* < 0.05 (two-sided) was considered statistically significant. Because of the exploratory nature of the study, no correction for multiple testing was applied, except for providing one example of a domain-specific Benjamini-Hochberg correction on all blood vitamin and minerals levels per group comparison. Analyses were performed with SAS software, version 9.4 (SAS Institute Inc, Cary, North Carolina, United States of America).

Normality was assessed by comparing the normal probability plot, the quantile-quantile plot (QQ plot), and the histogram of the (standardized) sample data to a normal probability curve. A non-parametric test was used if the normality assumption was not satisfied, except for the fatty acid composition parameters which were always tested non-parametrically. For paired comparisons with continuous data, paired *t*-test or non-parametric Wilcoxon signed rank test were performed. For paired comparisons with ordered categorical data, non-parametric Friedman test was used. For unpaired comparisons with continuous data, two sample *t*-test or non-parametric Wilcoxon rank sum test using Monte Carlo simulation was performed. Lastly, for unpaired comparisons with ordered categorical data, Wilcoxon rank sum test was used. For the two-sample *t*-tests, the pooled estimate for the error term for the t-statistic was used if equality of variance could be assumed, and if it could not, the degrees of freedom was adjusted using the Welch-Satterthwaite method.

Linear mixed models were performed for adjusted matched paired comparisons, while AN(C)OVA was performed for adjusted unpaired comparisons. Dependent variables and covariates for the adjusted comparisons were: serum vitamin D adjusted for estimated ultraviolet B (UVB) dose per month in Germany [derived from (20)]; serum glucose adjusted for diabetes medical history record; and serum cholesterol, serum vitamin D, and plasma coQ10 adjusted for use of HMG CoA (3-hydroxy-3-methylglutaryl coenzyme A) reductase inhibitors (statins). The interaction between vitamin D and UVB was also assessed with a model including an additional covariate x study group interaction term. An interaction term was considered significant at *p* < 0.10. To adjust for metformin use, the main group comparisons on blood levels of vitamin B6 and B12, folate, and homocysteine were also performed without stroke patients that used metformin (*N* = 11 in total).

A subgroup analysis were performed by dividing the stroke subjects based upon MNA-SF score (two groups, score 0–11 and score 12–14). HR subjects were divided in respective subgroups according to the subgroup status of their 1:1 matched stroke patients. Wilcoxon signed rank test was used for the paired comparison on subgroups.

## Results

### Subject characteristics

**Figure 2:**
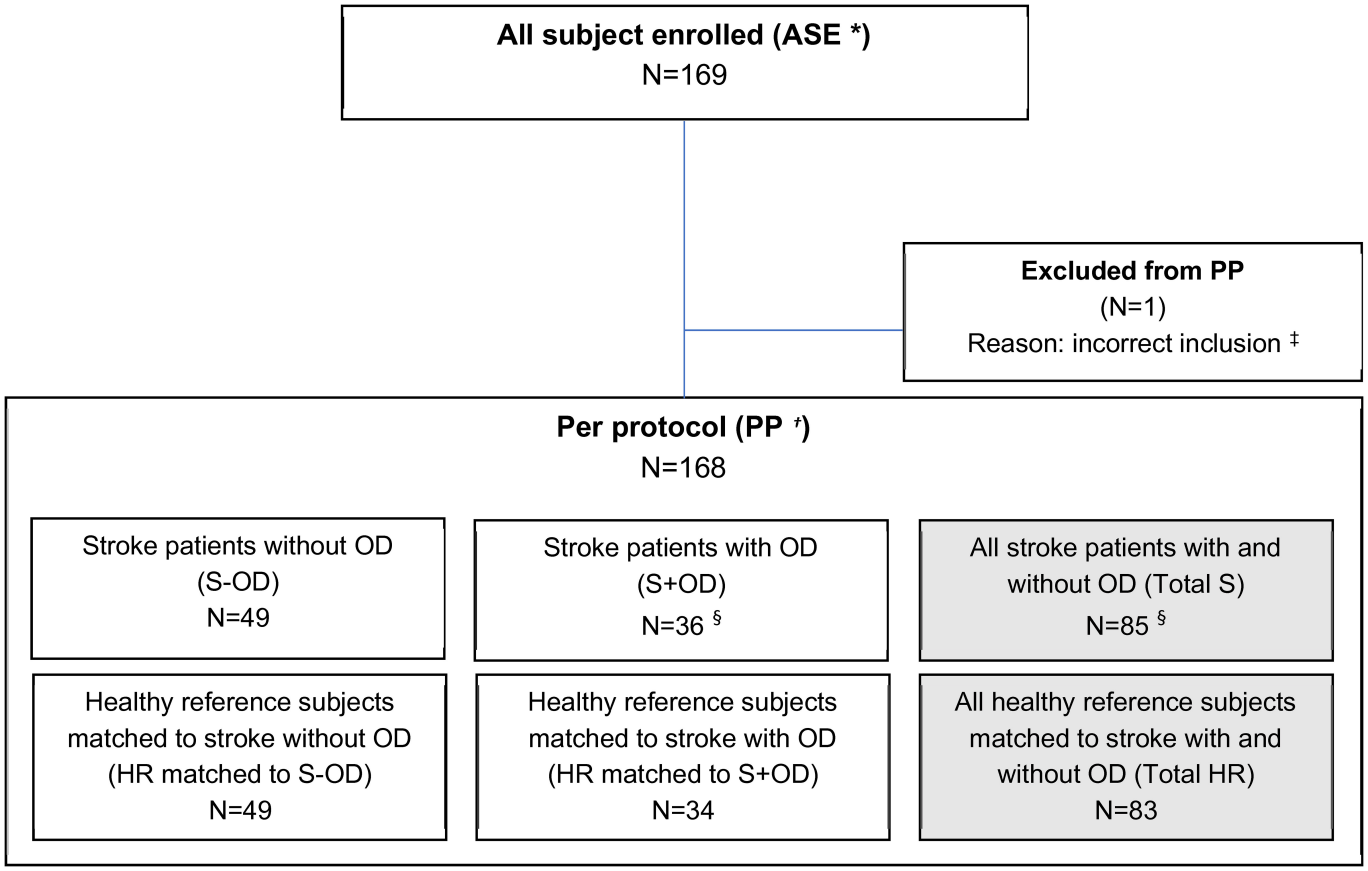
Trial profile. * ASE, All Subjects Enrolled population includes all subjects for whom informed consent was obtained and who passed the screening.^†^ PP, Per-Protocol population includes all subjects from the ASE population and excludes subjects with major protocol deviations.^‡^ Subject was 44 years which was an exclusion criterion for the stroke patients and thus regarded as major protocol deviation. ^§^ Two subjects in the S+OD group have not been matched to a HR subject due to the early termination of the study. These subjects obviously were only included in the unpaired comparisons (S-OD *vs*. S+OD) and not included in paired comparison.

Between January 4, 2018, and July 11, 2019, 169 participants of 177 screened were enrolled in the study. Because of the convincing results during the interim analyses, in combination with the exploratory nature of the study design, it was decided to stop the recruitment before completion of all 200 subjects. Recruitment was stopped after inclusion of 169 of the planned 200 subjects (see Figure 2). One subject was excluded for the per-protocol analysis because of a major protocol deviation.

Table 1 presents the characteristics of stroke subjects with and without OD and the matched HR subjects, including age, sex, BMI, and living situation before admission to the rehabilitation center. All subjects were Caucasians, except for 1 Asian in the S+OD group. Weight, height, socioeconomic status, educational status, current marital status, smoking status, and alcohol use are provided in the Supplementary material. For stroke subjects, stroke-specific characteristics are also presented in Table 1, including, time since stroke till the blood sample was taken, localization of stroke, and NIHSS score at hospital admittance, occurrence of tube feeding, medical ventilation, and medical complications during the rehabilitation phase. Time between stroke event and blood sample taken was comparable between the two stroke groups: on average 6 weeks, with a small variation in each group, whereas the inclusion criteria allowed was 2–12 weeks. NIHSS scores at admittance to the hospital were difficult to collect from the respective hospitals, resulting in high percentage of missing records. Records of relevant medical history were overall representative for ischemic stroke patients (data not shown). The most frequently prescribed concomitant medication in the stroke patients in the rehabilitation centers were: proton pump inhibitors, drugs for constipation, drugs for diabetes, antithrombotic agents, cardiovascular medication, psychoanaleptics, and psycholeptics (data not shown).

**Table 1:** Subject characteristics.

			***S-OD***	***HR matched to S-OD***	S+OD *	***HR matched to S+OD***	Total S *	***Total HR***
			***N = 49^‡^***	***N = 49***	***N = 36* ^†^**	***N = 34***	***N = 85^†^^‡^***	***N = 83***
Sex	Female	n (%)	18 (36.7)	18 (36.7)	6 (16.7)	5 (14.7)	24 (28.2)	23 (27.7)
Age	(Years)	mean (SD)	63.7 (6.7)	62.8 (6.8)	65.3 (6.2)	63.6 (5.8)	64.4 (6.5)	63.1(6.4)
		median (Q1-Q3)	63.0 (59.0-69.0)	63.0 (58.0–69.0)	65.0 (61.0–70.5)	62.0 (60.0–68.0)	64.0 (60.0–70.0)	63.0 (59.0–68.0)
BMI^†^	(kg/m^2^)	mean (SD)	27.6 (3.5)	26.0 (2.4)	26.0 (4.5)	26.6 (2.1)	26.9 (4.0)	26.2 (2.3)
Living situation	Institutionalized	n (%)	0 (0.0)	0 (0.0)	0 (0.0)	0 (0.0)	0 (0.0)	0 (0.0)
(Current for HR subjects. prior stroke for patients)	Home care		0 (0.0)	0 (0.0)	3 (8.3)	0 (0.0)	3 (3.5)	0 (0.0)
	Living independently (alone)		18 (36.7)	22 (44.9)	12 (33.3)	13 (38.2)	30 (35.3)	35 (42.2)
	Living independently (together)		31 (63.3)	27 (55.1)	21 (58.3)	21 (61.8)	52 (61.2)	48 (57.8)
Time after stroke till blood sampling^†^	Days	mean (SD)	40.4 (13.8)		41.3 (16.0)		40.8 (14.7)	
Localization of stroke	Right hemisphere	n (%)	27 (55.1)		17 (47.2)		44 (51.8)	
	Left hemisphere		14 (28.6)		7 (19.4)		21 (24.7)	
	Brainstem		7 (14.3)		8 (22.2)		15 (17.6)	
	Other		1 (2.0)		4 (11.1)		5 (5.9)	
NIHSS score at hospital admittance	0: no stroke symptom	n (%)	1 (2.0)		1 (2.8)		2 (2.4)	
	1–4::minor		11 (22.4)		4 (11.1)		15 (17.6)	
	5–15: moderate		12 (24.5)		8 (22.2)		20 (23.5)	
	16–20: moderate to severe		2 (4.1)		3 (8.3)		5 (5.9)	
	21–42: severe		0 (0.0)		0 (0.0)		0 (0.0)	
	Missing records		23 (46.9)		20 (55.6)		43 (50.6)	
Mechanical ventilation after stroke		n (%)	1 (2.0)		3 (8.3)		4 (4.7)	
Tube feeding after stroke (only allowed >2 weeks prior study entry)		n (%)	5 (10.2)		7 (19.4)		12 (14.1)	
Medical complications in rehabilitation phase^‡^	None	n (%)	46 (93.9)		20 (55.6)		66 (77.6)	
	Presence of urinary catheter		3 (6.1)		10 (27.8)		13 (15.3)	
	Infection urinary tract		1 (2.0)		4 (11.1)		5 (5.9)	
	Diarrhea		0 (0.0)		3 (8.3)		3 (3.5)	
	Pneumonia		0 (0.0)		1 (2.8)		1 (1.2)	
	Pressure ulcers		0 (0.0)		0 (0.0)		0 (0.0)	

^*^ including the two S+OD subjects who were not matched to HR subjects.

^†^ 1 subject (S+OD) had a missing BMI value; 3 subjects (S-OD) had missing records on time after stroke till blood sampling.

Missing records for NIHSS score are displayed in the table.

^‡^ Since a subject can have multiple medical complications, the numbers do not necessarily have to the total N.

BMI, body mass index; NIHSS, National Institutes of Health Stroke Scale; SD, standard deviation; Q1, Quartile 1; Q3, Quartile 3.

### Mini nutritional assessment–short form (MNA-SF)

MNA-SF screening score categories for each group is presented in Table 2. Risk of malnutrition and malnutrition were more often present in S-OD (44.9%) compared to their matched HR subjects (6.1%), with a significant difference in distribution over the MNA-SF score categories (*p* < 0.001). Likewise, (risk of) malnutrition was more often present in S+OD (64.7%) compared to matched HR subjects (0.0%, *p* < 0.001), as well as for Total S (53.0%) *vs*. Total HR (3.6%, *p* < 0.001). In turn, presence of OD after stroke was more often accompanied with (risk of) malnutrition *vs*. patients without OD (*p* = 0.013).

**Table 2:** MNA-SF score distribution per group.

		***S-OD***	***HR matched to S-OD***	***S+OD***	***HR matched to S+OD***	***Total S***	***Total HR***	S+OD *
**MNA-SF Score**		***N = 49***	***N = 49***	***N = 34***	***N = 34***	***N = 83***	***N = 83***	***N = 36***
Malnourished (score 0–7)	n (%)	6 (12.2)	0 (0.0)	9 (26.5)	0 (0.0)	15 (18.1)	0 (0.0)	10 (27.8)
At risk of malnutrition (score 8–11)		16 (32.7)	3 (6.1)	13 (38.2)	0 (0.0)	29 (34.9)	3 (3.6)	14 (38.9)
Normal nourished (score 12–14)		26 (53.1)	46 (93.9)	10 (29.4)	34 (100.0)	36 (43.4)	80 (96.4)	10 (27.8)
missing records		1 (2.0)	0 (0.0)	2 (5.9)	0 (0.0)	3 (3.6)	0 (0.0)	2 (5.6)
								
*p* value for difference in distribution over the MNA-SF score categories		***p*** **<** **0.001**^†^		***p*** **<** **0.001**^†^		***p*** **<** **0.001**^†^		***p*** **=** **0.013**^‡^ ***vs***. **S-OD**

^*^ including the two S+OD subjects who were not matched to HR subjects.

^†^ p value derived from Friedman's test.

^‡^ p value derived from the Wilcoxon rank sum test.

Bold p values are < 0.05.

MNA-SF, Mini Nutritional Assessment – Short Form.

### Fasted blood nutritional compounds and metabolites concentration

**Figure 3:**
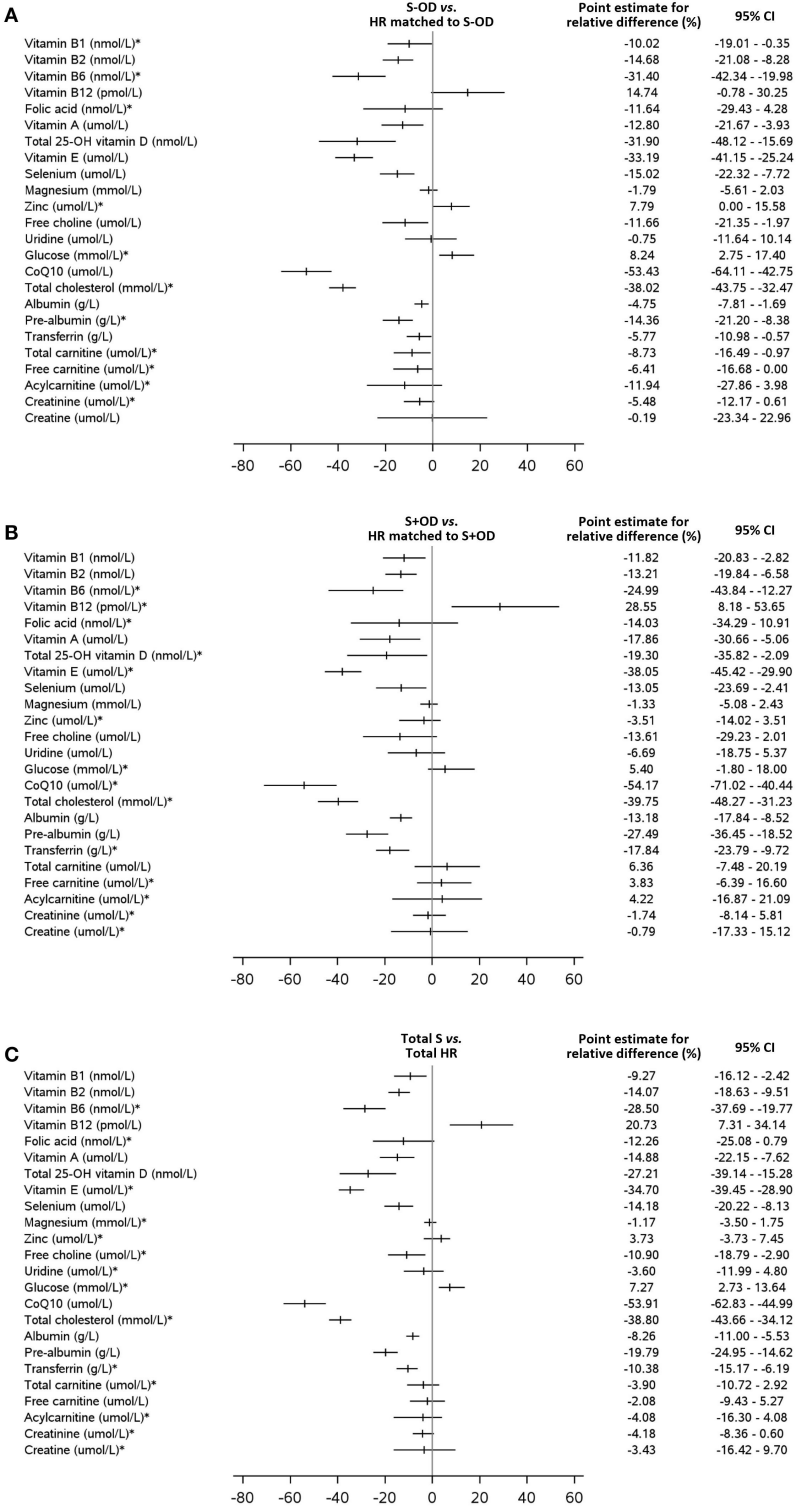
Relative differences (%) of blood concentrations of a selection of measured nutritional compounds and metabolites between stroke and HR groups. The relative difference is calculated as the difference between stroke patients [**(A)**: without OD, **(B)**: with OD, **(C)**: all stroke patients] and healthy reference subjects multiplied by 100 and divided by the mean of the healthy reference group. The point estimate (mean or median) for the relative difference and the 95% confidence interval (CI) are given for all blood parameters. No such relative differences plots are available for the comparison S-OD *vs*. S+OD. * the Hodges-Lehmann's median of the relative difference with the Hodges-Lehmann 95% CI (for two paired samples) is presented. For all other parameters the mean with the 95% CI based on Student distribution is used.

Tables 3A–C displays the whole blood, plasma, or serum concentrations for vitamin B1, B2, B6, A, total 25-OH vitamin D, and E, selenium, magnesium, zinc, choline, uridine, coQ10, glucose, cholesterol, albumin, pre-albumin, transferrin, total carnitine, free carnitine, acetyl carnitine, creatinine, creatine, CRP, and sodium, and serum osmolarity per subject group. Relative difference (%) of blood concentrations of a selection of measured nutritional compounds and metabolites between stroke and HR groups are plotted in Figures 3A–C.

**Table 3A:** Fasted blood nutritional compounds and metabolites concentration.

			***S-OD***	***HR matched to S-OD***	***S+OD***	***HR matched to S+OD***	***Total S***	***Total HR***	S+OD *
			***N = 49***	***N = 49***	***N = 34***	***N = 34***	***N = 83***	***N = 83***	***N = 36***
Vitamin B1	(nmol/L)	Mean (SD)	133.7 (37.5)	144.7 (27.2)	125.6 (27.3)	142.0 (23.3)	130.3 (33.7)	143.6 (25.5)	126.9 (27.1)
		Median (Q1–Q3)	130.5 (110.0–156.0)	146.0 (130.0–159.0)	128.0 (101.0–139.0)	138.5 (123.0–161.0)	130.0 (109.0-145.0)	143.0 (129.0-161.0)	130.0 (101.0-142.0)
		*p value*	**0.041** ^**§**^		**0.012** ^‡^		**0.009** ^‡^		0.697 ** (vs. S–OD)
Vitamin B2	(nmol/L)	Mean (SD)	242.0 (47.4)	280.3 (37.5)	232.7 (41.4)	270.5 (42.9)	238.1 (45.0)	276.3 (39.8)	234.7 (41.0)
		Median (Q1–Q3)	236.0 (218.0–259.0)	281.0 (255.0–297.0)	231.0 (207.0–254.0)	272.5 (239.0–305.0)	235.0 (209.0-259.0)	279.0 (247.0-302.0)	232.0 (207.0-262.0)
		*p value*	**<0.001** ^‡^		**<0.001** ‡		**<0.001** ‡		0.556 ** (vs. S–OD)
Vitamin B6	(nmol/L)	Mean (SD)	107.9 (241.3)	105.1 (31.3)	75.2 (22.5)	114.1 (51.4)	94.3 (184.6)	108.8 (40.7)	75.0 (22.0)
		Median (Q1–Q3)	66.5 (57.0–80.0)	96.0 (83.0–115.0)	71.0 (61.0–84.0)	97.0 (80.0–136.0)	67.0 (57.0-83.0)	96.0 (81.0-124.0)	71.0 (61.0-84.0)
		*p value*	**<0.001** ^**§**^		**<0.001** ^**§**^		**<0.001** ^**§**^		0.325 ** (vs. S–OD)
Vitamin B12	(pmol/L)	Mean (SD)	310.5 (113.6)	269.8 (82.0)	354.7 (152.5)	274.9 (118.8)	328.8 (132.1)	271.9 (98.1)	349.4 (151.4)
		Median (Q1–Q3)	298.0 (227.5–368.0)	274.0 (217.0–318.0)	311.5 (246.0–420.0)	273.5 (189.0–329.0)	307.5 (239.0-384.0)	274.0 (210.0-329.0)	311.5 (245.0-417.5)
		*p value*	0.062^‡^		**0.012** ^**§**^		**0.003** ^‡^		0.342 ** (vs. S–OD)
Folic acid †	(nmol/L)	Mean (SD)	17.3 (10.4)	18.7 (9.6)	16.7 (9.6)	19.2 (10.5)	17.1 (10.0)	18.9 (9.9)	18.3 (11.4)
		Median (Q1–Q3)	13.3 (10.9–20.1)	16.1 (11.7–23.5)	14.1 (9.5–19.6)	15.6 (12.5–24.8)	13.5 (10.6-19.6)	15.6 (11.9-24.2)	14.6 (9.9-22.5)
		*p value*	0.145 ^§^		0.282 ^§^		0.064 ^§^		0.999 ** (vs. S–OD)
Vitamin A	(μmol/L)	Mean (SD)	2.25 (0.51)	2.57 (0.50)	2.05 (0.68)	2.50 (0.47)	2.17 (0.59)	2.54 (0.48)	2.03 (0.68)
		Median (Q1–Q3)	2.22 (1.82–2.61)	2.54 (2.29–2.87)	2.03 (1.51–2.47)	2.39 (2.22–2.71)	2.18 (1.76-2.56)	2.43 (2.24-2.86)	2.02 (1.50-2.46)
		*p value*	**0.006** ^‡^		**0.008** ^‡^		**<0.001** ^‡^		0.086 || (vs. S–OD)
Total 25-OH vit D	(nmol/L)	Mean (SD)	42.5 (24.9)	63.2 (23.8)	46.0 (19.7)	57.5 (22.0)	44.0 (22.8)	60.9 (23.1)	45.4 (19.9)
		Median (Q1–Q3)	38.8 (20.8–57.4)	62.0 (46.1–72.4)	43.4 (29.7–60.6)	53.8 (40.0–69.0)	41.1 (27.0-60.6)	57.0 (44.0-72.4)	43.4 (29.4-59.9)
		*p value*	**<0.001** ^‡^		**0.022** ^**§**^		**<0.001** ^‡^		0.569 || (vs. S–OD)
Vitamin E	(μmol/L)	Mean (SD)	26.2 (6.4)	39.3 (8.9)	25.2 (7.5)	40.0 (13.2)	25.8 (6.8)	39.5 (10.8)	25.1 (7.5)
		Median (Q1–Q3)	26.5 (20.7–30.5)	38.0 (33.5–43.1)	23.5 (19.9–26.5)	37.1 (33.7–43.9)	24.7 (20.2-29.5)	37.9 (33.5-43.1)	23.5 (19.9-27.1)
		*p value*	**<0.001** ^‡^		**<0.001** ^**§**^		**<0.001** ^**§**^		0.468 || (vs. S–OD)
Selenium	(μmol/L)	Mean (SD)	0.87 (0.19)	1.01 (0.20)	0.92 (0.24)	1.06 (0.29)	0.89 (0.21)	1.03 (0.24)	0.91 (0.25)
		Median (Q1–Q3)	0.87 (0.77–0.95)	1.02 (0.85–1.12)	0.92 (0.82–1.01)	1.08 (0.82–1.23)	0.88 (0.80-0.98)	1.03 (0.85-1.15)	0.92 (0.80-1.02)
		*p value*	**<0.001** ^‡^		**0.018** ^‡^		**<0.001** ^‡^		0.358 || (vs. S–OD)
Magnesium	(mmol/L)	Mean (SD)	0.84 (0.10)	0.85 (0.05)	0.85 (0.08)	0.87 (0.07)	0.84 (0.09)	0.86 (0.06)	0.85 (0.09)
		Median (Q1–Q3)	0.86 (0.80–0.91)	0.86 (0.81–0.88)	0.86 (0.82–0.91)	0.87 (0.84–0.90)	0.86 (0.81-0.91)	0.86 (0.81-0.89)	0.86 (0.82-0.91)
		*p value*	0.352^‡^		0.477^‡^		0.478 ^§^		0.664 ** (vs. S–OD)
Zinc	(μmol/L)	Mean (SD)	14.0 (2.3)	12.8 (1.7)	13.5 (2.7)	14.3 (2.0)	13.8 (2.5)	13.4 (1.9)	13.5 (2.7)
		Median (Q1–Q3)	14.0 (12.0–16.0)	12.0 (12.0–14.0)	13.5 (12.0–15.0)	14.0 (13.0–15.0)	14.0 (12.0-15.5)	13.0 (12.0-15.0)	13.5 (12.0-15.0)
		*p value*	**0.025** ^**§**^		0.335 ^§^		0.310 ^§^		0.454 ** (vs. S–OD)

**Table 3B:** Fasted blood nutritional compounds and metabolites concentration.

			***S-OD***	***HR matched to S-OD***	***S+OD***	***HR matched to S+OD***	***Total S***	***Total HR***	S+OD *
			***N = 49***	***N = 49***	***N = 34***	***N = 34***	***N = 83***	***N = 83***	***N = 36***
Homocysteine	(μmol/L)	Mean (SD)	15.3 (5.7)	13.3 (3.8)	13.2 (4.6)	14.1 (6.0)	14.4 (5.3)	13.6 (4.8)	12.8 (4.7)
		Median (Q1–Q3)	13.8 (11.6–18.0)	12.4 (10.7–14.9)	12.9 (9.5–15.4)	14.0 (9.9–16.3)	13.5 (11.0–16.6)	13.0 (10.0–15.3)	12.1 (9.4–15.3)
		*p value*	0.116 ^§^		0.479^‡^		0.513 ^§^		**0.036** ** (vs. S–OD)
Free choline	(μmol/L)	Mean (SD)	8.27 (1.75)	9.35 (2.42)	8.39 (2.84)	9.72 (3.12)	8.32 (2.26)	9.50 (2.72)	8.33 (2.81)
		Median (Q1–Q3)	8.20 (7.03–8.93)	9.26 (7.54–10.50)	7.84 (6.23–9.75)	9.27 (7.80–11.00)	8.11 (6.53–9.10)	9.26 (7.69–10.70)	7.84 (6.06–9.70)
		*p value*	**0.019** ^‡^		0.085^‡^		**0.007** ^**§**^		0.577 ** (vs. S–OD)
Uridine	(μmol/L)	Mean (SD)	3.87 (0.92)	3.93 (1.05)	4.19 (1.63)	4.53 (1.40)	4.01 (1.28)	4.17 (1.24)	4.27 (1.63)
		Median (Q1–Q3)	3.85 (3.25–4.50)	4.00 (3.30–4.40)	3.65 (3.00–5.10)	4.60 (3.40–5.40)	3.80 (3.10–4.70)	4.00 (3.30–4.90)	3.75 (3.00–5.20)
		*p value*	0.890^‡^		0.267^‡^		0.342 ^§^		0.662 ** (vs. S–OD)
Glucose	(mmol/L)	Mean (SD)	6.34 (1.94)	5.46 (0.46)	6.28 (2.21)	5.56 (0.70)	6.32 (2.04)	5.50 (0.57)	6.33 (2.15)
		Median (Q1–Q3)	5.70 (5.20–6.65)	5.50 (5.10–5.80)	5.45 (5.00–6.70)	5.45 (5.10–5.80)	5.60 (5.20–6.70)	5.50 (5.10–5.80)	5.50 (5.05–6.75)
		*p value*	**0.004** ^**§**^		0.115 ^§^		**0.001** ^**§**^		0.670 ** (vs. S–OD)
Coenzyme Q10	(μmol/L)	Mean (SD)	0.741 (0.343)	1.565 (0.449)	0.669 (0.349)	1.481 (0.589)	0.712 (0.345)	1.531 (0.510)	0.688 (0.348)
		Median (Q1–Q3)	0.670 (0.460–0.910)	1.518 (1.218–1.891)	0.609 (0.499–0.720)	1.444 (1.142–1.887)	0.658 (0.474–0.821)	1.491 (1.175–1.891)	0.627 (0.499–0.799)
		*p value*	**<0.001** ^‡^		**<0.001** ^**§**^		**<0.001** ^‡^		0.500 ** (vs. S–OD)
Total cholesterol	(mmol/L)	Mean (SD)	3.46 (0.92)	5.50 (0.70)	3.50 (0.85)	5.64 (0.99)	3.48 (0.89)	5.55 (0.83)	3.56 (0.87)
		Median (Q1–Q3)	3.29 (2.75–4.03)	5.37 (5.17–6.06)	3.38 (3.07–3.74)	5.58 (4.86–6.18)	3.33 (2.92–3.89)	5.44 (4.99–6.12)	3.40 (3.08–3.82)
		*p value*	**<0.001** ^**§**^		**<0.001** ^**§**^		**<0.001** ^**§**^		0.415 ** (vs. S–OD)
Albumin	(g/L)	Mean (SD)	37.6 (3.7)	39.5 (2.5)	34.6 (4.4)	39.8 (3.1)	36.3 (4.3)	39.6 (2.7)	34.5 (4.3)
		Median (Q1–Q3)	37.8 (36.0–39.5)	39.6 (37.9–41.0)	35.0 (33.0–37.7)	39.5 (37.4–42.0)	36.9 (34.8–38.9)	39.6 (37.8–41.5)	35.0 (33.0–37.7)
		*p value*	**0.003** ^‡^		**<0.001** ^‡^		**<0.001** ^‡^		**<0.001** **(vs. S–OD)
Pre-albumin	(g/L)	Mean (SD)	0.252 (0.043)	0.292 (0.044)	0.223 (0.061)	0.306 (0.051)	0.240 (0.053)	0.298 (0.047)	0.223 (0.060)
		Median (Q1–Q3)	0.251 (0.218–0.280)	0.290 (0.265–0.317)	0.220 (0.177–0.260)	0.292 (0.264–0.336)	0.240 (0.210–0.275)	0.290 (0.264–0.327)	0.220 (0.177–0.260)
		*p value*	**<0.001** ^**§**^		**<0.001** ^‡^		**<0.001** ^‡^		**0.017 # (vs. S–OD)**
Transferrin	(g/L)	Mean (SD)	2.36 (0.38)	2.49 (0.28)	2.04 (0.39)	2.52 (0.45)	2.23 (0.41)	2.51 (0.36)	2.05 (0.38)
		Median (Q1–Q3)	2.30 (2.11–2.52)	2.50 (2.30–2.70)	2.00 (1.70–2.20)	2.40 (2.20–2.90)	2.20 (1.98–2.50)	2.50 (2.30–2.70)	2.00 (1.70–2.25)
		*p value*	**0.030** ^‡^		**<0.001** ^**§**^		**<0.001** ^**§**^		**<0.001** **(vs. S–OD)

**Table 3C:** Fasted blood nutritional compounds and metabolites concentration.

			***S–OD***	***HR matched to S–OD***	***S+OD***	***HR matched to S+OD***	***Total S***	***Total HR***	S+OD *
			***N = 49***	***N = 49***	***N = 34***	***N = 34***	***N = 83***	***N = 83***	***N = 36***
Total carnitine	(μmol/L)	Mean (SD)	46.1 (12.2)	51.5 (8.3)	53.7 (14.5)	51.0 (10.8)	49.2 (13.6)	51.3 (9.4)	52.5 (15.4)
		Median (Q1–Q3)	48.5 (41.5–54.0)	52.0 (46.0–58.0)	54.0 (45.0–59.0)	50.5 (46.0–57.0)	50.0 (42.0–56.0)	51.0 (46.0–58.0)	53.0 (45.0–59.0)
		*p value*	**0.031** ^**§**^		0.356^‡^		0.208 ^§^		**0.033** **(vs. S–OD)
Free carnitine	(μmol/L)	Mean (SD)	35.9 (8.9)	39.0 (7.2)	41.1 (11.1)	39.1 (7.6)	38.0 (10.1)	39.0 (7.3)	40.2 (12.1)
		Median (Q1–Q3)	36.5 (31.0–41.0)	39.0 (33.0–44.0)	42.0 (35.0–46.0)	40.0 (31.0–43.0)	37.0 (32.0–43.0)	40.0 (33.0–44.0)	41.0 (32.0–46.0)
		*p value*	0.063 ^§^		0.437 ^§^		0.575^‡^		0.053 ** (vs. S–OD)
Acylcarnitine	(μmol/L)	Mean (SD)	10.9 (4.4)	12.6 (3.8)	12.6 (5.2)	11.9 (4.9)	11.6 (4.8)	12.3 (4.3)	12.3 (5.2)
		Median (Q1–Q3)	11.0 (7.0–14.0)	12.0 (10.0–14.0)	12.0 (10.0–14.0)	12.5 (8.0–15.0)	11.5 (9.0–14.0)	12.0 (10.0–15.0)	12.0 (10.0–14.0)
		*p value*	0.109 ^§^		0.752 ^§^		0.333 ^§^		0.348 ** (vs. S–OD)
Creatinine	(μmol/L)	Mean (SD)	78.0 (20.8)	82.2 (16.8)	90.5 (43.4)	86.0 (9.6)	83.2 (32.5)	83.7 (14.4)	88.8 (43.1)
		Median (Q1–Q3)	74.5 (65.0–86.0)	80.0 (73.0–92.0)	84.0 (73.0–96.0)	84.5 (80.0–94.0)	78.5 (67.0–92.0)	84.0 (76.0–94.0)	82.5 (73.0–95.0)
		*p value*	0.057 ^§^		0.645 ^§^		0.089 ^§^		0.062 ** (vs. S–OD)
Creatine	(μmol/L)	Mean (SD)	34.3 (21.5)	34.7 (20.1)	33.7 (28.6)	31.7 (19.3)	34.0 (24.5)	33.5 (19.7)	35.0 (28.3)
		Median (Q1–Q3)	28.8 (19.2–41.0)	27.0 (17.8–44.6)	24.9 (18.7–35.7)	26.2 (18.2–46.9)	26.6 (19.2–36.7)	26.6 (17.8–45.6)	26.2 (19.1–37.7)
		*p value*	0.987^‡^		0.848 ^§^		0.574 ^§^		0.668 ** (vs. S–OD)
C-reactive protein †	(mg/L)	Mean (SD)	4.41 (6.27)	1.73 (1.81)	13.69 (36.30)	1.86 (2.19)	8.26 (24.10)	1.79 (1.96)	13.07 (35.35)
		Median (Q1–Q3)	1.95 (0.87–5.32)	1.00 (0.70–1.90)	4.10 (1.00–11.80)	1.20 (0.90–1.80)	2.80 (0.90–7.10)	1.10 (0.70–1.90)	4.10 (1.00–11.68)
		*p value*	**0.001** ^**§**^		**<0.001** ^**§**^		**<0.001** ^**§**^		0.185 ** (vs. S–OD)
Sodium	(mmol/L)	Mean (SD)	139.80 (2.63)	138.82 (1.57)	140.72 (2.39)	139.72 (2.06)	140.18 (2.56)	139.19 (1.83)	140.85 (2.38)
		Median (Q1–Q3)	140.10 (138.70–141.45)	138.60 (137.60–139.70)	141.00 (139.60–142.00)	139.40 (138.40–141.40)	140.75 (139.00–142.00)	139.10 (137.90–140.30)	141.00 (139.80–142.30)
		*p value*	**0.008** ^**§**^		0.086^‡^		**0.006** ^‡^		**0.043** **(vs. S–OD)
Osmolality	(mOsm/kg)	Mean (SD)	295.19 (6.85)	293.18 (5.56)	294.47 (6.32)	293.09 (6.23)	294.89 (6.60)	293.14 (5.81)	294.83 (6.34)
		Median (Q1–Q3)	295.00 (292.50–298.00)	292.00 (290.00–297.00)	294.50 (291.00–298.00)	292.50 (289.00–298.00)	295.00 (292.00–298.00)	292.00 (289.00–297.00)	295.00 (291.00–298.75)
		*p value*	**0.046** ^**§**^		0.342 ^§^		**0.032** ^**§**^		0.714 ** (vs. S–OD)

Missing values (e.g. due to lack of volume) ranged between 1 and a maximum of 6 cases per parameter within all 168 subjects.

^*^ including the two S+OD subjects who were not matched to HR subjects.

^†^ 5 subjects (2 S-OD, 1 HR matched to S-OD, and 2 HR matched to S+OD) had serum folate concentration above the upper limit of detection. In these cases, the upper limit of detection value was taken; 7 subjects (6 HR matched to S-OD, and 1 S+OD) had C-Reactive Protein concentration below the lower limit of detection. In these cases, values were replaced by half of the lower limit of detection.

‡ p value derived from a paired-t-test.

^§^ p value derived from a Wilcoxon signed rank test.

^||^ p value derived from two sample t-test with the Pooled method (equal variances).

^#^ p value derived from two sample t-test with the Satterthwaite method (unequal variances).

^**^ p value derived from a Wilcoxon rank sum test using Monte Carlo simulation.

Bold p values are <0.05.

Q1, Quartile 1; Q3, Quartile 3; SD, standard deviation.

Supplement use was not an exclusion criterion for the stroke subjects, except for vitamin B12 injections. Supplement use was recorded as concomitant medication and was relatively low. Excluding the supplement users from the analyses on blood levels did not reveal other insights as with their inclusion.

#### Vitamins and minerals concentration in blood plasma or serum

Concentrations of whole blood vitamins B1 (*p* = 0.041, *p* = 0.012, and *p* = 0.009, respectively), B2 (all *p* < 0.001), and B6 (all *p* < 0.001), serum vitamin A (*p* = 0.006, *p* = 0.008, and *p* < 0.001, respectively), E (all *p* < 0.001), total 25-OH vitamin D (*p* < 0.001, *p* = 0.022, and *p* < 0.001, respectively), and plasma selenium (*p* < 0.001, *p* = 0.018, and *p* < 0.001, respectively) were lower in S-OD, S+OD, and Total S subjects as compared to their matched HR subjects; no significant differences were found when comparing S+OD *vs*. S-OD subjects. Relative differences between Total S and Total HR subjects were: −9% for vitamin B1, −14% for B2, −28% for B6, −15% for A, −27% for D, −35% for E, and −14% for selenium. Differences in vitamin D concentration between the stroke and HR groups remained significant after adjusting for estimated potential UVB dose (see Supplementary material).

Considerable variation was observed in serum folate concentration, and the four group comparisons did not reveal significant differences. Serum vitamin B12 concentration was higher in the S+OD (*p* = 0.012) and Total S subjects (*p* = 0.003, relative difference: +21%) compared to their matched HR subjects, albeit S-OD *vs*. matched HR and S-OD *vs*. S+OD did not significantly differ. When stroke patients that used metformin were excluded (*N* = 9 for S-OD and *N* = 2 for S+OD) from the main comparisons for vitamin B6 and B12, and folate, a higher absolute blood concentration was observed for all three B-vitamins, especially in the S-OD group (see Supplementary material), without affecting observed significances.

Plasma zinc and serum magnesium concentrations were not significantly different in the four group comparisons, except for zinc being higher in S-OD *vs*. their matched HR subject (*p* = 0.025).

Correcting the different comparisons for multiple testing on the domain of all vitamins and minerals, most of the differences remained significant (see Supplementary material).

#### Other nutritional compounds and metabolites in blood plasma or serum

Plasma homocysteine concentration was not significantly different for S-OD, S+OD, and Total S *vs*. matched HR subjects, however homocysteine was higher in S-OD compared to S+OD (*p* = 0.036). The later difference was not maintained when metformin users were excluded. Excluding metformin users resulted in lower homocysteine concentration in the stroke groups (see Supplementary material).

Plasma choline concentration was lower for S-OD (*p* = 0.019) and Total S (*p* = 0.007) *vs*. their matched HR subjects, with a relative difference for Total S *vs*. Total HR of −11%. Free choline was not significantly different for S+OD *vs*. matched HR subjects and S-OD *vs*. S+OD. No differences were observed for serum uridine concentration in the four comparisons.

Serum glucose concentration was higher in S-OD (*p* = 0.004) and Total S (*p* = 0.001, relative difference: +7%) *vs*. their matched HR subjects, with no significant differences in the other two group comparisons. The significant difference of glucose between the Total S and Total HR group disappeared after adjusting for presence of diabetes (see Supplementary material).

Plasma coQ10 (all *p* < 0.001) and serum total cholesterol (all *p* < 0.001) concentrations were lower for S-OD, S+OD, and Total S *vs*. matched HR subjects. No significant differences were observed for S-OD *vs*. S+OD. Relative differences between Total S and Total HR subjects were: −54% for coQ10 and −39% for cholesterol. Differences in coQ10 and cholesterol concentrations between the Total S and Total HR group remained significant after adjusting for statin use (see Supplementary material).

Serum albumin (*p* = 0.003, *p* < 0.001, and *p* < 0.001, respectively), pre-albumin (all *p* < 0.001), and transferrin (*p* = 0.030, *p* < 0.001, and *p* < 0.001, respectively) concentrations were lower for S-OD, S+OD, and Total S *vs*. their matched HR subjects. Higher levels for albumin (*p* < 0.001), pre-albumin (*p* = 0.017), and transferrin (*p* < 0.001) were observed for S-OD *vs*. S+OD. Relative differences between Total S and Total HR subjects were: −8% for albumin, −20% for pre-albumin, and −10% for transferrin.

Plasma total carnitine concentration was lower in S-OD *vs*. their matched HR subjects (*p* = 0.031) and *vs*. S+OD (*p* = 0.033), with no significant differences for S+OD *vs*. matched HR subjects and for Total S *vs*. Total HR. Free carnitine and acetyl carnitine and serum creatinine and creatine concentrations did not differ between the compared groups.

Significantly higher serum CRP concentration was observed for S-OD, S+OD, and Total S *vs*. their matched HR subjects (all *p* ≤ 0.001), with a relative difference for Total S *vs*. Total HR of +137%. CRP concentration was not significantly different between S-OD and S+OD.

Serum sodium concentration (*p* = 0.008 and *p* = 0.006, respectively) and osmolarity (*p* = 0.046 and *p* = 0.032, respectively) were higher in the S-OD and Total S *vs*. their matched HR subjects, albeit S+OD *vs*. matched HR did not differ significantly. Furthermore, a lower sodium concentration was observed in S-OD *vs*. S+OD (*p* = 0.043). Osmolarity was not significantly different between S-OD and S+OD.

### Fasted blood total lipid fatty acid composition

Tables 4A, B present an overview of the most relevant fatty acids in plasma in quantitative concentrations (A), and in erythrocytes as weight percentage of total fatty acid content (B). In addition, the sums are displayed for the saturated fatty acids (SFA), monounsaturated fatty acids (MUFA), omega-3 polyunsaturated fatty acids (n3 PUFA), omega-6 PUFA (n6 PUFA), total PUFA, and the total of all fatty acids (only applicable for quantitative concentrations).

**Table 4A:** Fasted plasma total lipid fatty acid composition.

			***S-OD***	***HR matched to S-OD***	***S+OD***	***HR matched to S+OD***	***Total S***	***Total HR***	S+OD *
			***N = 49***	***N = 49***	***N = 34***	***N = 34***	***N = 83***	***N = 83***	***N = 36***
Plasma fatty	C18:2 n-6 (LA)	Mean (SD)	705.7 (186.7)	1052.7 (214.0)	616.1 (181.2)	1043.8 (236.5)	667.6 (188.5)	1049.1 (222.1)	615.5 (176.1)
acids (mg/L)		Median (Q1-Q3)	666.0 (580.6-812.6)	1036.1 (915.1-1162.0)	552.1 (494.6-687.8)	1032.7 (909.0–1193.8)	625.8 (533.0–754.3)	1035.3 (909.0–1189.9)	553.6 (497.9–681.5)
		*p value*	**<0.001** ^**§**^		**<0.001** ^**§**^		**<0.001** ^**§**^		**0.004** **(***vs***. **S–OD)**
	C18:3 n-3 (ALA)	Mean (SD)	12.53 (5.24)	20.90 (25.40)	9.26 (2.72)	18.96 (9.08)	11.14 (4.62)	20.10 (20.29)	9.16 (2.68)
		Median (Q1–Q3)	11.47 (9.26–14.18)	14.92 (11.65–20.87)	8.59 (7.59–10.59)	16.34 (12.54–23.37)	10.31 (8.21–13.03)	15.93 (11.89–22.93)	8.39 (7.48–10.31)
		*p value*	**0.001** ^**§**^		**<0.001** ^**§**^		**<0.001** ^**§**^		**<0.001** **(***vs***. **S–OD)**
	C20:4 n-6 (AA)	Mean (SD)	213.55 (51.66)	245.75 (55.65)	212.80 (50.89)	216.35 (53.17)	213.23 (51.01)	233.70 (56.23)	215.46 (50.88)
		Median (Q1–Q3)	212.04 (184.34–248.29)	247.87 (197.99–285.74)	209.80 (180.39–247.17)	217.55 (181.99–244.58)	211.42 (181.09–248.06)	229.05 (193.96–272.54)	214.12 (181.09–255.97)
		*p value*	**0.019** ^**§**^		0.497 ^§^		**0.026** ^**§**^		0.779 ** (*vs*. S–OD)
	C20:5n-3 (EPA)	Mean (SD)	15.76 (5.70)	29.28 (17.16)	12.88 (4.92)	29.21 (16.97)	14.54 (5.54)	29.25 (16.98)	13.64 (5.77)
		Median (Q1–Q3)	14.61 (11.95–18.43)	25.19 (16.30–37.79)	11.59 (9.38–16.39)	23.75 (15.14–41.57)	13.42 (10.47–17.86)	25.19 (15.58–40.40)	11.96 (9.61–17.03)
		*p value*	**<0.001** ^**§**^		**<0.001** ^**§**^		**<0.001** ^**§**^		0.051 ** (*vs*. S–OD)
	C22:6n-3 (DHA)	mean (SD)	42.85 (14.98)	49.15 (18.98)	42.71 (12.54)	52.73 (24.85)	42.79 (13.91)	50.61 (21.51)	43.22 (12.75)
		median (Q1–Q3)	40.53 (34.10–50.54)	46.31 (35.44–60.15)	43.08 (34.11–50.26)	47.12 (34.81–67.98)	40.73 (34.10–50.40)	47.01 (35.37–62.03)	43.08 (34.19–50.73)
		*p value*	**0.036** ^**§**^		0.053 ^§^		**0.005** ^**§**^		0.815 ** (*vs*. S–OD)
	Total SFA	Mean (SD)	988.3 (216.8)	1,134.7 (362.6)	946.2 (231.8)	1,227.1 (482.9)	970.4 (222.9)	1,172.6 (415.8)	953.8 (238.0)
		Median (Q1–Q3)	970.7 (800.0–1,124.1)	1,045.4 (953.0–1,251.6)	863.6 (797.7–1,007.1)	1,116.7 (1,007.2–1,324.5)	904.0 (798.9–1,098.5)	1,086.3 (953.0–1,261.6)	863.6 (793.8–1,038.7)
		*p value*	**0.021** ^**§**^		**0.003** ^**§**^		**<0.001** ^**§**^		0.290 ** (*vs*. S–OD)
	Total MUFA	Mean (SD)	918.1 (263.6)	985.1 (429.8)	911.9 (261.2)	1,087.0 (587.3)	915.4 (261.0)	1,026.8 (499.5)	914.3 (265.7)
		Median (Q1–Q3)	912.0 (713.0–1,099.8)	880.7 (768.5–1,028.1)	862.1 (751.8–1,030.5)	926.9 (825.2–1,178.4)	877.2 (717.6–1,090.9)	915.4 (778.7–1,076.3)	862.1 (726.1–1,042.1)
		*p value*	0.480 ^§^		0.076 ^§^		0.085 ^§^		0.797 ** (*vs*. S–OD)
	Total PUFA	mean (SD)	1064.7 (228.2)	1488.0 (279.2)	963.1 (232.3)	1452.0 (292.1)	1021.5 (234.0)	1473.3 (283.4)	968.6 (227.0)
		median (Q1–Q3)	1,050.2 (919.4–1,211.7)	1,477.9 (1,300.3–1,588.4)	909.6 (801.2–,1029.1)	1,462.9 (1,248.9–1,635.0)	955.5 (857.3–1,140.8)	1,473.6 (1,284.3–1,605.7)	923.0 (810.6–1,043.8)
		*p value*	**<0.001** ^**§**^		**<0.001** ^**§**^		**<0.001** ^**§**^		**0.016** **(***vs***. **S–OD)**
	Total n3 PUFA	Mean (SD)	83.5 (22.5)	114.6 (50.9)	75.6 (19.9)	116.0 (45.4)	80.2 (21.6)	115.2 (48.4)	77.1 (21.0)
		Median (Q1–Q3)	80.2 (71.3–95.2)	104.2 (79.2–129.0)	73.5 (62.8–87.4)	111.4 (83.8–146.4)	76.2 (66.1–92.0)	105.7 (82.0–142.6)	74.6 (62.9–88.1)
		*p value*	**<0.001** ^**§**^		**<0.001** ^**§**^		**<0.001** ^**§**^		0.126 ** (*vs*. S–OD)
	Total n6 PUFA	Mean (SD)	978.8 (216.4)	1,370.0 (255.0)	885.0 (217.3)	1,332.2 (269.6)	938.9 (220.4)	1,354.5 (260.1)	889.0 (211.8)
		Median (Q1–Q3)	946.7 (829.1–1,126.3)	1336.3 (1,207.5–1,487.8)	829.1 (742.1–943.9)	1,345.0 (1,169.0–1,485.1)	878.8 (781.6–1,050.9)	1,336.3 (1,195.6–1,487.8)	848.1 (744.2–960.5)
		*p value*	**<0.001** ^**§**^		**<0.001** ^**§**^		**<0.001** ^**§**^		**0.021** **(***vs***. **S–OD)**
	Total FA	Mean (SD)	2,987.0 (647.9)	3,628.9 (1,004.5)	2,837.2 (660.4)	3,787.1 (1,306.1)	2,923.3 (653.4)	3,693.7 (1132.8)	2,853.1 (664.2)
		Median (Q1–Q3)	2,942.9 (2,456.3–3,440.3)	3,349.7 (3,084.6–3,994.3)	2,645.5 (2,465.0–2,989.2)	3,531.1 (3,166.3–4,056.5)	2,740.3 (2,460.7–3,317.0)	3,431.7 (3,127.0–4,056.5)	2,645.5 (2,460.3–2,993.7)
		*p value*	**<0.001** ^**§**^		**<0.001** ^**§**^		**<0.001** ^**§**^		0.327 ** (*vs*. S–OD)

**Table 4B:** Fasted erythrocyte total lipid fatty acid composition.

			***S-OD***	***HR matched to S-OD***	***S+OD***	***HR matched to S+OD***	***Total S***	***Total HR***	S+OD *
			***N = 49***	***N = 49***	***N = 34***	***N = 34***	***N = 83***	***N = 83***	***N = 36***
Erythrocyte fatty	C18:2 n-6 (LA)	Mean (SD)	10.73 (2.05)	14.55 (1.98)	9.30 (1.54)	14.90 (2.45)	10.10 (1.96)	14.69 (2.18)	9.24 (1.60)
acids (weight % of total		Median (Q1–Q3)	10.48 (9.26–11.41)	14.55 (13.03–15.62)	9.12 (8.03–10.23)	15.05 (13.52–16.72)	9.81 (8.59–10.88)	14.71 (13.08-15.76)	9.12 (7.96-10.27)
fatty acids)		*p value*	**<0.001** ^**§**^		**<0.001** ^**§**^		**<0.001** ^**§**^		**0.001** **(***vs***. **S–OD)**
	C18:3 n-3 (ALA)	Mean (SD)	0.13 (0.06)	0.26 (0.26)	0.07 (0.05)	0.24 (0.10)	0.10 (0.06)	0.25 (0.21)	0.07 (0.05)
		Median (Q1–Q3)	0.12 (0.10–0.17)	0.21 (0.17–0.27)	0.08 (0.05–0.11)	0.21 (0.17–0.29)	0.10 (0.07–0.13)	0.21 (0.17-0.28)	0.08 (0.06-0.10)
		*p value*	**<0.001** ^**§**^		**<0.001** ^**§**^		**<0.001** ^**§**^		**<0.001** **(***vs***. **S–OD)**
	C20:4 n-6 (AA)	Mean (SD)	11.15 (1.21)	10.44 (1.13)	11.56 (1.25)	9.65 (1.44)	11.33 (1.24)	10.12 (1.32)	11.57 (1.22)
		Median (Q1–Q3)	11.08 (10.25–12.18)	10.65 (9.90–11.05)	11.35 (10.76–12.52)	9.77 (9.01–10.57)	11.29 (10.45–12.23)	10.26 (9.44-10.89)	11.37 (10.79-12.52)
		*p value*	**0.004** ^**§**^		**<0.001** ^**§**^		**<0.001** ^**§**^		0.180 ** (*vs*. S–OD)
	C20:5n-3 (EPA)	Mean (SD)	0.61 (0.25)	0.83 (0.38)	0.54 (0.17)	0.85 (0.40)	0.58 (0.22)	0.84 (0.39)	0.55 (0.17)
		Median (Q1–Q3)	0.58 (0.43–0.73)	0.76 (0.59–1.06)	0.54 (0.43–0.62)	0.84 (0.47–1.14)	0.56 (0.43–0.67)	0.79 (0.54-1.08)	0.56 (0.44-0.64)
		*p value*	**<0.001** ^**§**^		**<0.001** ^**§**^		**<0.001** ^**§**^		0.442 ** (*vs*. S–OD)
	C22:6n-3 (DHA)	Mean (SD)	3.01 (0.81)	2.56 (0.63)	3.05 (0.59)	2.66 (0.73)	3.03 (0.72)	2.60 (0.67)	3.02 (0.58)
		Median (Q1–Q3)	2.92 (2.50–3.38)	2.51 (2.14–2.89)	2.99 (2.52–3.51)	2.72 (2.21–3.19)	2.96 (2.51–3.42)	2.52 (2.14-3.07)	2.94 (2.51-3.43)
		*p value*	**0.002** ^**§**^		**0.048** ^**§**^		**<0.001** ^**§**^		0.824 ** (*vs*. S–OD)
	Total SFA	Mean (SD)	43.6 (1.8)	42.2 (1.7)	45.0 (2.0)	42.3 (2.5)	44.2 (2.0)	42.2 (2.0)	45.1 (2.0)
		Median (Q1–Q3)	43.6 (42.5–44.5)	42.1 (40.7–43.5)	45.0 (43.6–46.2)	42.4 (40.7–44.3)	44.2 (43.1–45.1)	42.3 (40.7-43.8)	45.1 (43.7-46.6)
		*p value*	**<0.001** ^**§**^		**<0.001** ^**§**^		**<0.001** ^**§**^		**<0.001** **(***vs***. **S–OD)**
	Total MUFA	Mean (SD)	21.7 (1.5)	21.1 (2.0)	21.6 (1.7)	21.8 (2.2)	21.7 (1.6)	21.3 (2.1)	21.6 (1.7)
		Median (Q1–Q3)	21.6 (20.7–22.6)	20.9 (19.7–21.9)	21.3 (20.4–22.9)	21.4 (20.6–22.8)	21.4 (20.6–22.8)	21.1 (19.8-22.2)	21.3 (20.4-22.9)
		*p value*	0.165 ^§^		0.841 ^§^		0.282 ^§^		0.646 ** (*vs*. S–OD)
	Total PUFA	Mean (SD)	31.7 (2.3)	34.1 (1.9)	30.4 (2.2)	33.5 (2.6)	31.1 (2.3)	33.9 (2.2)	30.3 (2.2)
		Median (Q1–Q3)	32.0 (30.9–33.1)	34.1 (32.6–35.4)	30.3 (28.6–32.4)	33.3 (31.1–35.6)	31.5 (29.2–32.7)	34.1 (32.3-35.4)	30.3 (28.6-32.3)
		*p value*	**<0.001** ^**§**^		**<0.001** ^**§**^		**<0.001** ^**§**^		**0.005** **(***vs***. **S–OD)**
	Total n3 PUFA	Mean (SD)	5.2 (1.2)	5.1 (1.2)	5.1 (0.8)	5.1 (1.2)	5.2 (1.0)	5.1 (1.2)	5.1 (0.8)
		Median (Q1–Q3)	5.1 (4.6–5.7)	5.0 (4.4–5.6)	5.1 (4.5–5.6)	4.9 (4.3–6.0)	5.1 (4.5–5.6)	4.9 (4.3-5.7)	5.1 (4.5-5.6)
		*p value*	0.543 ^§^		0.728 ^§^		0.872 ^§^		0.773 ** (*vs*. S–OD)
	Total n6 PUFA	Mean (SD)	26.4 (2.2)	29.0 (2.0)	25.3 (2.0)	28.3 (2.8)	25.9 (2.2)	28.7 (2.4)	25.2 (2.0)
		Median (Q1–Q3)	26.7 (25.3–27.4)	28.9 (27.8–30.2)	25.3 (23.6–26.8)	27.9 (26.4–30.4)	26.1 (24.2–27.3)	28.7 (27.1-30.4)	25.3 (23.4-26.7)
		*p value*	**<0.001** ^**§**^		**<0.001** ^**§**^		**<0.001** ^**§**^		**0.014** **(***vs***. **S–OD)**

Missing values (e.g. due to lack of volume) for plasma analyses were 3 and for erythrocytes analyses were 11 within all 168 subjects.

*including the two S+OD subjects who were not matched to HR subjects.

^§^p value derived from a Wilcoxon signed rank test.

**p value derived from a Wilcoxon rank sum test using Monte Carlo simulation.

Bold p values are <0.05.

AA, Arachidonic acid; ALA, alpha-linolenic acid; DHA, docosahexaenoic acid; EPA, eicosapentaenoic acid; LA, linoleic acid; MUFA, monounsaturated fatty acids; n3, omega-3; n6, omega-6; PUFA, polyunsaturated fatty acids; Q1, Quartile 1; Q3, Quartile 3; SD, standard deviation; SFA, saturated fatty acids.

Fatty acid composition in both plasma and erythrocytes differed considerably between the stroke and HR groups.

Plasma concentrations of eicosapentaenoic acid (EPA), linoleic acid (LA), alpha-linolenic acid (ALA), SFA, n3 PUFA, n6 PUFA, total PUFA, and total fatty acid (all *p* < 0.05, see table for exact *p* values), but not MUFA, were lower in S-OD, S+OD and Total S *vs*. their matched HR subjects. Similar results were found for docosahexaenoic acid (DHA) concentrations in plasma, with lower concentrations in S-OD (*p* = 0.036) and Total S (*p* = 0.005) *vs*. their matched HR subjects, except for the lower concentration in S+OD *vs*. matched HR not being significant (*p* = 0.053). Arachidonic acid (AA) concentration was lower in S-OD (*p* = 0.019) and Total S (*p* = 0.026) *vs*. their matched HR subjects, with no differences for S+OD *vs*. matched HR. In addition, plasma LA, ALA, n6 PUFA, and total PUFA were higher in S-OD *vs*. S+OD (all *p* < 0.05). Plasma concentrations of AA, EPA, DHA, MUFA, n3 PUFA, and total fatty acids did not significantly differ between S-OD and S+OD. Relative differences between Total S and Total HR subjects for plasma EPA and DHA were: −47 and −14%, respectively.

Erythrocyte level of EPA, LA, ALA, n6 PUFA, total PUFA (all *p* < 0.001), but not MUFA and n3 PUFA, as a percentage of total fatty acid content, were lower in S-OD, S+OD and Total S *vs*. their matched HR subjects. In contrast, erythrocyte DHA, SFA, and AA (all *p* < 0.05, see table for exact *p* values) percentage of total fatty acids were significantly higher in S-OD, S+OD, and Total S subjects compared to their matched HR subjects. In addition, erythrocyte LA, ALA, n6 PUFA, and total PUFA were higher in S-OD *vs*. S+OD (all *p* < 0.05), and SFA was lower in S-OD *vs*. S+OD (*p* < 0.001). Erythrocyte level of AA, EPA, DHA, MUFA, and n3 PUFA did not significantly differ between S-OD and S+OD. Relative differences between Total S and Total HR subjects for erythrocyte EPA and DHA were: −14 and +13%, respectively.

### Reported nutritional intake

Reported energy and macronutrient intake are summarized here, where a more complete overview of nutritional intake is presented in the Supplementary material. Results are presented for the Total S and Total HR groups only. Cautions interpretation is required because of limitation of the methodology, which were accepted during study design setting. For example, a 1-day food diary instead of 3-day food diary was recorded (see Discussion and conclusions for further elaboration).

Total S subjects reported a lower total energy intake per day *vs*. Total HR (mean (SD) 1,857 (550) *vs*. 2,168 (597) kcal/day, *p* < 0.001). Likewise, total energy intake per day per kg body weight (BW) was reportedly lower in Total S *vs*. Total HR (mean (SD) 23.4 (7.5) *vs*. 27.5 (7.7) kcal/kg BW/day, *p* < 0.001).

The lower reported energy intake in the Total S subjects is reflected by lower reported intake of all macronutrients. Total S subjects reported lower intake in g/kg BW/day of fat (mean (SD) 1.01 (0.41) *vs*. 1.20 (0.46), *p* = 0.004), protein (mean (SD) 0.93 (0.33) *vs*. 1.14 (0.39), *p* < 0.001), carbohydrate (mean (SD) 2.50 (0.89) *vs*. 2.82 (1.08), *p* = 0.048), and fiber (mean (SD) 0.24 (0.10) *vs*. 0.27 (0.12), *p* = 0.011) compared to their matched HR subjects.

Reported water intake (g/kg BW/day) was also lower in Total S subjects compared to their matched HR subjects (median (Q1-Q3) of 28.4 (22.1–36.9) *vs*. 32.7 (26.2–43.8), *p* = 0.004).

### Quality of life and activities of daily living

Table 5 displays the reported scores of the EQ-5D-5L and Barthel Index questionnaires. Scores on separate categories of both questionnaires are more elaborately presented in the Supplementary material.

**Table 5:** EQ-5D-5L index and VAS and Barthel index scores.

		**S-OD**	**HR matched to S-OD**	**S+OD**	**HR matched to S+OD**	**Total S**	**Total HR**	S+OD *
		***N = 49***	***N = 49***	***N = 34***	***N = 34***	***N = 83***	***N = 83***	***N = 36***
EQ-5D-5L index value †	Mean (SD)	0.80 (0.29)	0.98 (0.04)	0.43 (0.33)	0.98 (0.05)	0.65 (0.35)	0.98 (0.04)	0.44 (0.33)
	Median (Q1-Q3)	0.92 (0.81–1.00)	1.00 (1.00–1.00)	0.26 (0.23–0.68)	1.00 (1.00–1.00)	0.81 (0.26–1.00)	1.00 (1.00–1.00)	0.27 (0.23–0.71)
	*p* value	**<0.001** ^**§**^		**<0.001** ^‡^		**<0.001** ^**§**^		**<0.001** **(***vs***. **S–OD)**
EQ-5D-5L VAS score †	Mean (SD)	65.4 (21.3)	91.9 (7.4)	51.9 (18.8)	91.8 (6.9)	60.0 (21.3)	91.8 (7.1)	52.7 (18.6)
	Median (Q1-Q3)	70 (50–83)	93 (90–98)	50 (40–65)	90 (90–99)	60 (49–78)	92 (90–99)	50 (40–65)
	*p* value	**<0.001** ^**§**^		**<0.001** ^**§**^		**<0.001** ^**§**^		**0.003** **(***vs***. **S–OD)**
Barthel index score	Mean (SD)	87.2 (15.2)	100.0 (0.0)	49.6 (29.7)	100.0 (0.0)	71.8 (29.0)	100.0 (0.0)	51.4 (30.1)
	Median (Q1-Q3)	95 (80–100)	100 (100–100)	45 (20–75)	100 (100–100)	80 (55–100)	100 (100–100)	52.5 (22.5–77.5)
	*p* value	**<0.001** ^**§**^		**<0.001** ^**§**^		**<0.001** ^**§**^		**<0.001** **(***vs***. **S–OD)**

^*^ including the two S+OD subjects who were not matched to HR subjects.

† 1 subject (S-OD) had a missing record on EQ-5D-5L VAS score and 2 subjects (S+OD) had missing record on EQ-5D-5L Index Value and VAS score.

^‡^ p value derived from a paired-t-test.

§ p value derived from a Wilcoxon signed rank test.

^**^ p value derived from a Wilcoxon rank sum test using Monte Carlo simulation.

Bold p values are < 0.05.

EQ-5D-5L, EuroQol 5-dimensions 5-level; Q1, Quartile 1; Q3, Quartile 3; SD, standard deviation; VAS, visual analog scale.

#### EQ-5D-5L index value

S-OD, S+OD, and Total S subjects (all *p* < 0.001) had reported a lower quality of life index value compared to their matched HR subjects. Reported quality of life was higher in S-OD compared to S+OD (*p* < 0.001).

S+OD subjects reported more often “extreme problems” in the five separate categories of the index value than S-OD subjects, with mobility: 50.0% vs. 14.3%, self-care: 16.7 *vs*. 2.0%, and usual activities: 38.9 vs. 10.2%. For pain/discomfort and anxiety/depression, S+OD subjects reported less often “no problems” compared to S-OD subjects (66.7 vs. 73.5%, and 58.3 vs. 69.4%, respectively). Most Total HR subjects reported “no problems” for mobility (97.6%), self-care (100.0%), usual activities (97.6%), pain/discomfort (80.7%), and anxiety/depression (100%).

#### EQ-5D-5L VAS score

S-OD, S+OD, and Total S subjects (all *p* < 0.001) reported their health state lower compared to their matched HR subjects. S-OD subjects reported their health state to be higher compared to S+OD subjects (median 70 *vs*. 50, *p* = 0.003).

#### Barthel index

S-OD, S+OD, and Total S subjects (all *p* < 0.001) experienced more problems with activities of daily living compared to their matched HR subjects. In turn, S-OD subjects experienced less problems with daily activities than S+OD subjects (*p* < 0.001).

S+OD subjects reported less often independency on the separate categories of activities of daily living compared to S-OD subjects, with bowels: 61.1 vs. 100.0%, bladder: 47.2 vs. 93.9%, grooming: 61.1 vs. 95.9%, toilet use: 47.2 vs. 87.8%, feeding: 36.1 vs. 95.9%, transfers: 27.8 vs. 87.8%, mobility: 22.2 vs. 61.2%, dressing: 25.0 vs. 79.6%, stairs: 13.9 vs. 49.0%, and bathing: 13.9 vs. 59.2%. Total HR subjects reported the maximal score for all 10 categories, meaning that they were fully independent in all these 10 activities of daily living.

### Subgroup analysis

A subgroup analysis was performed on most blood markers, EQ-5D-5L index value and VAS score, and the Barthel index (see Supplementary material). All stroke subjects, with and without OD, were divided into two subgroups based upon their MNA-SF score, with *N* = 44 for score 0–11 (malnourished or at risk of malnutrition) and *N* = 36 for score 12–14 (normal nourished) and were compared to their matched HR subjects. Overall, no notable differences between the subgroups comparisons and the overall comparisons were found for the blood markers, the EQ-5D-5L index value and VAS score, and the Barthel index. A noteworthy difference is however serum osmolality, which was only higher in the normal nourished stroke subgroup *vs*. matched HR subjects (*p* < 0.001).

### Safety parameters

In total, 25 medical events were reported for stroke subjects and none in the HR subjects. The most frequently reported medical event was worsening of arterial hypertension. Most of the reported medical events were mild with a short duration. All medical events started before the blood sampling, and therefore probably none of the medical events were related to study-specific procedures. No serious adverse events have been reported.

## Discussion

The current findings indicate a highly impaired nutritional status in ischemic stroke patients with and without oropharyngeal dysphagia during sub-acute inpatient rehabilitation. More than half of the stroke patients displayed (risk of) malnutrition, with higher prevalence in patient with OD *vs*. without OD (MNA-SF scores). Fasted blood concentrations of vitamins B1, B2, B6, A, D, and E, selenium, choline, CoQ10, albumin, pre-albumin, transferrin, DHA, EPA, LA, and ALA were all significantly lower in stroke compared to age- and sex-matched matched HR subjects, irrespective of OD status. Stroke patients had a poorer hydration status as reflected by higher blood sodium and osmolality levels *vs*. HR subjects. Inflammatory marker CRP was highly increased in both stroke patients with and without OD *vs*. HR subjects. Reported energy, macronutrient, and water intake were lower in stroke patients *vs*. HR. As expected, QoL and ADL were significantly lower in stroke *vs*. HR, with OD scoring worse than non-OD patients.

The current study provides novel insights to existing knowledge, firstly because it provides a comprehensive nutritional evaluation of sub-acute ischemic stroke patients with data on presence or risk of malnutrition, a large set of measured blood nutritional compounds and metabolites, and indications on nutritional intake. Secondly, stroke patients with or without OD were included, and group comparisons between presence and absence of OD on all nutritional, ADL, and QoL parameters were investigated. Lastly, this study included age- and sex matched HR subjects and employed pair matched analysis to increase statistical precision, because it is known that age and sex affect nutritional status, especially blood nutritional compound levels.

Fifty-three percent of the stroke patients had (a risk of) malnutrition as reflected by MNA-SF scores. More stroke patients with OD (64.7%) than patients without OD (44.9%) had (a risk of) malnutrition. Although reported malnutrition prevalence varies widely across studies, overall a high prevalence is reported, with malnutrition being most common in stroke patients with dysphagia. In their systematic review, Foley et al. found prevalence ranging between 8 and 49% for malnutrition in stroke patients during both the acute and rehabilitation stages (8). In addition, the odds of being malnourished were higher among stroke patients with dysphagia as compared to stroke patients without dysphagia. In a recent meta-analysis, Huppertz et al. reported a pooled prevalence of 37% for malnutrition in the sub-acute phase and an overall prevalence range for dysphagia between 6 and 88% (2). The included studies in these reviews most commonly did not stratify for dysphagia as in the current study, and therefore do not directly report prevalence of malnutrition in stroke patients with or without dysphagia [e.g., (21)].

Blood concentrations of many of the measured nutritional compounds and metabolites were significantly lower in stroke patients, both with and without OD. Relative changes ranged from −55% to occasional higher levels for some compounds up to +25% compared to the HR subjects. Hence, the presence, direction, and magnitude of the differences between stroke and HR subjects varied between the individual nutritional compounds and metabolites. Overall, measured levels did not differ between stroke patients with and without OD.

The measured blood nutritional markers and metabolites in the current study were selected based on their relevance in stroke, either because they are involved in stroke-relevant processes or previously found to be associated with functional outcome. Numerous previous studies have provided data on nutritional compound concentrations in blood of stroke patients (meta-analysis, Broersen et al., in preparation). Most studies have been conducted in the acute or in the chronic phase, while studies in patients in the rehabilitation phase are scarcer. Previous studies on blood levels in stroke also usually do not provide information on patients' OD status. The most extensive investigated nutritional compounds in stroke are vitamin D, B12 and folate. Fewer studies were done on zinc, magnesium, selenium, vitamins A, B6, and E, and DHA and EPA blood levels after stroke, and none or only one or two on B1, B2, choline, uridine, and coQ10. The presence and order of magnitude of the difference in concentration differ among studies which is probably founded in differences in study population characteristics (age, race, country, stroke subgroup, etc.), sample size, state of fasting, analytical methods, and confounder adjustment.

In line with current findings, previous studies (repeatedly) showed lower blood levels of vitamin A [e.g., (22)], D [e.g., (14)], and E [e.g., (23)], selenium [e.g., (24)], DHA and EPA [e.g., (15)], and choline (25) compared to controls. Vitamin B6 blood levels were lower (also when metformin users were excluded) in stroke patients *vs*. HR subjects, whereas previous studies vary in outcomes with some studies even reporting higher levels in patients *vs*. controls (24). This is one of the first studies reporting on blood levels of vitamin B1 and B2 in stroke patients compared to controls and both vitamins appeared lower in stroke. Current results did not reveal differences in blood levels of magnesium, although lower levels were reported in previous studies [e.g., (26)]. Contrary to the lower serum B12 and folate levels in stroke patients as commonly found in previous studies [e.g., (13, 27)], the current stroke patients had similar blood concentrations of folate and higher concentration of vitamin B12 compared to HR subjects. An explanation for the contradictory finding is lacking; future studies should investigate the direction of vitamin B12 effects further. Homocysteine is frequently reported to be higher in stroke patients [e.g., (28)], as would be expected considering the usually reported lower levels of folate and B12 (and B6 in the current study). In the current study however, similar levels were found in the stroke groups as compared to their matched HR subjects, with or without excluding metformin users. Metformin is known to affect blood folate, B6, B12, and homocysteine status (29, 30) and therefore comparisons were also performed while excluding metformin users. Despite the relatively low number of stroke patients that used metformin (in total *N* = 11), excluding them resulted in evident higher vitamin B6 and B12, and folate levels and lower homocysteine levels. Hence, the observed differences between the stroke patients and healthy subjects therefore became greater (vitamin B12) or smaller (vitamin B6).

HMG CoA reductase inhibitors (statins) are known to reduce coQ10 blood levels (31), and these lower levels are linked to statin-induced-myopathy (32). In the current stroke patients, plasma coQ10 levels were 54% lower than in HR subjects and even remained significant after adjusting for statin use (for Total S vs. Total HR), indicating a stroke-specific reduction of coQ10, as previously also suggested (33). Statin use most probably explains the lower serum cholesterol level observed in the stroke patients compared to HR subjects, although after adjusting for statin use the difference remained significant. The observed higher serum glucose levels in stroke patients compared to HR subjects was clearly linked to diabetes, since the significant difference disappeared after adjusting for the presence of diabetes. The higher glucose levels were observed despite the glucose lowering medication used in diabetic stroke patients. Stroke patients had lower levels of serum transferrin, pre-albumin, and albumin compared to HR subjects, and in turn were lower in stroke patients with OD *vs*. patients without OD. These three visceral proteins are an indication for malnutrition (34) and are in line with the reported (risk of) malnutrition with the MNA-SF.

An increase in the inflammatory marker CRP has repeatedly been observed in both dysphagia (35) and stroke (36) and is associated with poor clinical outcome (37). In the current, study CRP was also highly increased in stroke patients, especially in patients with OD.

Subgroup analyses, with stroke patients divided into two subgroups (malnourished or at risk of malnutrition and normal nourished) compared to their matched HR subjects, showed that almost all differences or equivalences found between the stroke subgroups *vs*. HR were irrespective of the patients' nutritional status (MNA-SF). This might imply disease-specific changes in blood nutrient concentrations that go beyond overall protein-energy malnutrition.

The insufficiencies of certain nutrition compounds in blood can be caused by several stroke-specific and general factors that affect nutrient intake, uptake, and metabolism. For example, dysphagia, impaired consciousness, tube feeding, and motor deficits reducing oral intake; (poly)medication and comorbidities affecting nutrient uptake and metabolism; and stroke-related ongoing inflammatory processes, gut dysbiosis, and brain pathology and recovery process affecting metabolism (3, 4, 38–41). Also, insufficiencies can already exist prior to stroke and malnutrition is indicated as a risk factor for stroke (4). Whether the basal metabolic rate is increased in the acute or subacute period after stroke remains unclear (42). In addition, total energy requirement also depends on the physical activity levels and varies highly among stroke patients as they might be bedridden or follow high intensity rehabilitation physical activities.

Since stroke patients admitted to a rehabilitation center suffer from a variety of conditions including cognitive and motor impairments, a considerable part of the patients would have difficulties or would not have been able to keep a food diary. Therefore, the involvement of nursing staff, therapists, family, and visitors of the patients was needed. For practical reasons, it was chosen to collect nutritional intake data over 1 day instead of 3 days that would probably have yielded more accurate data. Furthermore, it turned out that stroke patients and study staff had difficulties completing the diaries according to the protocol. Consequently, the results should be interpreted with caution. Nonetheless, lower energy intake was reported by stroke patients (with and without OD) compared to matched HR subjects (23.4 *vs*. 27.5 kCal/kg body weight). Assuming that resting energy expenditure is not elevated in stroke patients in rehabilitation compared to healthy reference subjects, and considering rehabilitation physical activities, total energy requirements for stroke patients in rehabilitation could be estimated to be equal to healthy elderly, with requirements around 30 kCal/kg body weight per day. The lower energy intake in stroke patients of the current study would be roughly 78% of the average energy requirement. This is in line with previous studies on nutritional intake in stroke reporting intakes to be too low, varying around 60-90% the daily estimated energy requirements in the subacute and chronic phase (43–45). The lower reported energy intakes in the current study were also reflected by lower reported intakes of macronutrients. Stroke patients reported water intake of approximately 80% of their HR subjects and might lead to a poorer hydration status. This was also reflected in markers for dehydration, i.e., higher sodium concentration and osmolality in stroke patients *vs*. HR subjects. These findings are in line with an Australian study reporting that stroke patients without dysphagia in rehabilitation consumed 67% of their estimated daily requirement for fluid intake, with 44% being dehydrated measured by blood urea nitrogen to creatinine ratio (46). The lower energy and macronutrient intake in stroke patients in the current study might be related to the stroke and/or dysphagia. Also, lower physical activity level in physically impaired stroke patients might drive toward lower nutritional intake. Furthermore, the differences in nutritional intake between stroke patients and HR subjects may have been larger than the current findings due to a reporting bias: HR subjects might be inclined to underreport their nutritional intake, because they could choose to only report the healthy foods and drinks; whereas stroke patients might tend to overreport the food consumed since the meals offered could have been reported, rather than the food really consumed.

Not surprisingly and in line with previous observations (47–49), stroke patients in the current study reported having a lower quality of life and experienced more problems with activities of daily living compared HR subjects, and OD further worsens these deficiencies. Combined OD and (risk of) malnutrition seems to be particularly detrimental for QoL and ADL (data not shown).

More males than females were included in the stroke patient group with OD (83%) and without OD (63%). The higher percentages of males in this study population are probably due to the inclusion criteria of age ≥ 50 and ≤ 75 years. Since on average more females get strokes when they are older, fewer females could have been eligible for inclusion in this study. The age limit of 75 years was taken, because nutritional status is known to be affected by age. In addition, difficulties were expected to find enough willing HR subjects above this age. Recruiting eligible stroke patients, and especially stroke patient with OD, turned out to be challenging. The upper age limit substantially hampered the inclusion rate of stroke patients. In addition, many stroke patients with OD were not eligible either because of early recovery from dysphagia or because dysphagia severity required tube feeding.

With a mean BMI of 26.9 of the stroke patients and 26.2 of the HR subjects, more than 75% of both groups were overweight or obese. Considering that more than half of the stroke patients in the current study were (at risk of being) malnourished, clearly demonstrates that overweight can coexist with malnutrition. Therefore, overweight stroke patients should not be disregarded by deeming them well nourished.

Several study limitations were identified. Firstly, the in- and exclusion criteria for the stroke and HR groups might have resulted in more homogeneous and more specifically characterized groups than in clinical practice. Secondly, screening for OD with BODS could have resulted in more misclassifications than with instrumental assessments for dysphagia (e.g., fiberoptic endoscopic evaluation of swallowing [FEES]). BODS was chosen to minimize the patient burden and because this test was standard clinical practice in the two rehabilitation centers. Furthermore, males were overrepresented, which could have influenced group differences, even though HR subjects were sex-matched to the stroke patients. In addition, as explained above, there were challenges collecting the nutritional intake data and therefore these should be interpreted with caution as well. Then, results were not adjusted for multiple testing due to the exploratory nature of the study, hence all reported differences are preliminary and need confirmation in further studies. Additionally, concomitant medication and comorbidities could act as confounders or outcome modifiers, especially because the medical history of the HR group was not recorded, and medication use heavily differed between stroke patients and HR. Some of the concomitant medication and comorbidities were also exclusion criteria for HR subjects, but not for stroke patients. Therefore, additional statistical comparisons were adjusted for some of the most apparent concomitant medication and comorbidities. Lastly, all parameters were measured once (i.e. on average 6 weeks after onset) which gives only information from one time point after stroke, and therefore, no conclusions can be drawn as to causality or change over time. Nevertheless, at the moment of assessment, the nutritional status of stroke patients was heavily impaired compared to matched HR subjects, and a consistent pattern in concentration of nutritional compounds and metabolites was seen in stroke patients *vs*. matched HR subjects.

The importance of nutritional management after stroke is well recognized by most relevant guidelines, which generally recommend early screening for dysphagia and malnutrition and the consideration of oral nutritional supplements for patients who are malnourished or at risk of malnourishment (50–55). These recommendations are supported by a recent meta-analysis concluding that impaired nutritional status on admission is associated with poor functional recovery and increased mortality (56). The lower levels of many nutritional compounds as found in current study may result in suboptimal substrate availability for numerous structural and functional recovery processes after stroke. For example, nutritional compounds serve as antioxidants, co-enzymes in metabolic pathways, and precursors for neurotransmitters and synaptic membranes. Evidence in turn suggests that micronutrient supplementation may help in stroke rehabilitation as measured by various outcomes measures (42, 57). Timely intervention is important, as the window for effective rehabilitation therapies is short; improvement in functioning is most evident within the first 3 months after stroke onset (58). Despite the guidelines and emerging evidence, nutritional supplementation seems not to be consistently considered as an effective contributor to neurorehabilitation and functional recovery after stroke.

Nutritional status was highly impaired in sub-acute stroke patients admitted to rehabilitation centers. Interestingly, blood levels of specific nutritional compounds and metabolites were similarly lower in stroke patients with or without OD *vs*. HR, even though patients with OD were more likely to be malnourished. These results underline that it is important to screen for nutritional impairments in every stroke patient, either with or without OD. Readily accessible laboratory testing of blood nutritional compounds, such as vitamin B6, D, and E, could therefore be considered. Whether tailored nutritional supplementation after a stroke could support rehabilitation should be investigated in future trials.

## Data availability statement

The datasets presented are available upon reasonable request. Any requests will be reviewed against compliance with ethical, scientific, regulatory, and legal requirements. Requests to access the datasets should be directed to NvW, nick.van-wijk@danone.com.

## Ethics statement

The studies involving human participants were reviewed and approved by Ethics Review Committee Ärztekammer Nordrhein in Germany (identifier: MPR16TA07987). The patients/participants provided their written informed consent to participate in this study.

## Author contributions

ML, DR, MS, and TS-W were involved in study concept and design. BS, MS, and TS-W were involved in selection of patients, study conduct, and acquisition of data. NvW, DR, CAvdB, and ML were involved in statistical planning and analysis. NvW and CAvdB drafted the manuscript which all authors critically reviewed. All authors contributed to interpretation of data and results and approved the final version of the manuscript, including the authorship list.
