# Supplementary material for: Evident lower blood levels of multiple nutritional compounds and highly prevalent malnutrition in sub-acute stroke patients with or without dysphagia
Source: Front Neurol. 2023 Jan 10;13:1028991. doi: 10.3389/fneur.2022.1028991 (PMC9872791; doi:10.3389/fneur.2022.1028991)
Supplement: Supplementary file 1 [file Table_1.docx]

# Supplementary Material

#### Table S1. List of inclusion and exclusion criteria.

|  | Stroke group | Age- and sex-matched healthy reference group |
| --- | --- | --- |
| Inclusion criteria | 1. Diagnosis of ischemic stroke | 1. Body Mass Index (BMI): ≥ 20 and < 30 kg/m2 |
|  | 2. Time after stroke: ≥ 2 and ≤ 12 weeks | 2. Written informed consent |
|  | 3. Inpatient in a stroke rehabilitation center | |
|  | 4. Age ≥ 50 and ≤ 75 years | |
|  | 5. Written informed consent * | |
| Exclusion criteria | 1. Diagnosis of hemorrhagic stroke | 1. Having a special diet, e.g. receiving oral nutritional support or tube feed, or having a dysphagia-adapted diet, vegan diet or ketogenic diet |
|  | 2. Known history of progressive neurological disorders (e.g. Parkinson’s disease, MS) | 2. Known diabetes mellitus type 2 |
|  | 3. Dysphagia not related to stroke | 3. Known disorders of the gastrointestinal (GI) tract, including coeliac disease |
|  | 4. Receiving chemo- or radiotherapy within 1 year prior to entry into the study | 4. Known history of cardiovascular or cerebrovascular disease, e.g. myocardial infarct, stroke or transient ischemic attack |
|  | 5. Receiving tube feeding or has been receiving tube feeding within 2 weeks prior to entry into the study | 5. Use of anti-hypertensive or cholesterol- or triglyceride-lowering drugs |
|  | 6. Current prescription of vitamin injection | 6. Hospital admittance (with overnight stay) within 6 months prior to entry into the study |
|  | 7. Investigator’s uncertainty about the ability to adhere to the protocol requirements because of the condition of the patient | 7. Receiving chemo- or radiotherapy within 1 year prior to entry into the study |
|  | 8. Participation in any other studies involving investigational or marketed products within 6 weeks prior to entry into the study | 8. Decrease in appetite and/or food intake within 4 weeks prior to entry into the study |
|  |  | 9. Known weight loss of >3 kg in the last 3 months |
|  |  | 10. Participation in weight loss diet within 3 months prior to entry into the study |
|  |  | 11. Current moderate or heavy alcohol use (>14 consumptions per week for females or >21 consumptions per week for males), moderate or heavy smoking (≥10 cigarettes or ≥5 cigars/pipes per day) or drug abuse to the opinion of the investigator |
|  |  | 12. Blood donation within 4 weeks prior to entry into the study |
|  |  | 13. Investigator's uncertainty about the willingness or ability of the subject to comply with the protocol requirements |
|  |  | 14. Participation in any other clinical study involving investigational or marketed products within 6 weeks prior to entry into the study |

* Part of the population under investigation were incapacitated and therefore the legal representative of the patient was informed about the study and gave informed consent.

#### Table S2. Additional subject characteristics.

|  |  |  | ***S-OD*** | ***HR matched to***  ***S-OD*** | ***S+OD **** | ***HR matched to S+OD*** | ***Total S **** | ***Total HR*** |
| --- | --- | --- | --- | --- | --- | --- | --- | --- |
|  |  |  | *N = 49 ‡* | *N = 49* | *N = 36* † | *N = 34* | *N = 85 †‡* | *N = 83* |
| **Socioeconomic status** | Employed | n (%) | 23 (46.9%) | 21 (42.9%) | 14 (38.9%) | 12 (35.3%) | 37 (43.5%) | 33 (39.8%) |
|  | Unemployed |  | 2 (4.1%) | 3 (6.1%) | 1 (2.8%) | 5 (14.7%) | 3 (3.5%) | 8 (9.6%) |
|  | Retired |  | 24 (49.0%) | 25 (51.0%) | 21 (58.3%) | 17 (50.0%) | 45 (52.9%) | 42 (50.6%) |
|  | Disabled |  | 0 (0.0%) | 0 (0.0%) | 0 (0.0%) | 0 (0.0%) | 0 (0.0%) | 0 (0.0%) |
| **Educational level** | Primary education not completed | n (%) | 1 (2.0%) | 0 (0.0%) | 0 (0.0%) | 0 (0.0%) | 1 (1.2%) | 0 (0.0%) |
|  | Primary education |  | 9 (18.4%) | 5 (10.2%) | 9 (25.0%) | 1 (2.9%) | 18 (21.2%) | 6 (7.2%) |
|  | Secondary education |  | 29 (59.2%) | 30 (61.2%) | 20 (55.6%) | 24 (70.6%) | 49 (57.6%) | 54 (65.1%) |
|  | Tertiary education |  | 10 (20.4%) | 14 (28.6%) | 7 (19.4%) | 9 (26.5%) | 17 (20.0%) | 23 (27.7%) |
| **Current marital status** | Married (includes living together) | n (%) | 31 (63.3%) | 23 (46.9%) | 22 (61.1%) | 17 (50.0%) | 53 (62.4%) | 40 (48.2%) |
|  | Widowed |  | 5 (10.2%) | 2 (4.1%) | 4 (11.1%) | 1 (2.9%) | 9 (10.6%) | 3 (3.6%) |
|  | Divorced |  | 8 (16.3%) | 12 (24.5%) | 7 (19.4%) | 8 (23.5%) | 15 (17.6%) | 20 (24.1%) |
|  | Never married |  | 5 (10.2%) | 12 (24.5%) | 3 (8.3%) | 8 (23.5%) | 8 (9.4%) | 20 (24.1%) |
| **Smoking status** | No | n (%) | 34 (69.4%) | 45 (91.8%) | 21 (58.3%) | 30 (88.2%) | 55 (64.7%) | 75 (90.4%) |
|  | Yes |  | 15 (30.6%) | 4 (8.2%) | 15 (41.7%) | 4 (11.8%) | 30 (35.3%) | 8 (9.6%) |
|  | Light smoker |  | 4 (26.7%) | 4 (100.0%) | 5 (33.3%) | 4 (100.0%) | 9 (30.0%) | 8 (100.0%) |
|  | Moderate smoker |  | 2 (13.3%) | 0 (0.0%) | 6 (40.0%) | 0 (0.0%) | 8 (26.7%) | 0 (0.0%) |
|  | Heavy smoker |  | 9 (60.0%) | 0 (0.0%) | 4 (26.7%) | 0 (0.0%) | 13 (43.3%) | 0 (0.0%) |
| **Alcohol use** | No | n (%) | 19 (38.8%) | 12 (24.5%) | 10 (27.8%) | 7 (20.6%) | 29 (34.1%) | 19 (22.9%) |
|  | Yes |  | 30 (61.2%) | 37 (75.5%) | 26 (72.2%) | 27 (79.4%) | 56 (65.9%) | 64 (77.1%) |
|  | ≤ 7 units per week |  | 28 (93.3%) | 34 (91.9%) | 25 (96.2%) | 16 (59.3%) | 53 (94.6%) | 50 (78.1%) |
|  | 8 - 14 units per week |  | 2 (6.7%) | 3 (8.1%) | 1 (3.8%) | 11 (40.7%) | 3 (5.4%) | 14 (21.9%) |
|  | 15 - 21 units per week |  | 0 (0.0%) | 0 (0.0%) | 0 (0.0%) | 0 (0.0%) | 0 (0.0%) | 0 (0.0%) |
|  | > 21 units per week |  | 0 (0.0%) | 0 (0.0%) | 0 (0.0%) | 0 (0.0%) | 0 (0.0%) | 0 (0.0%) |
| **Body weight**† | (kg) | mean (SD) | 82.27 (13.99) | 78.39 (11.08) | 78.18 (15.69) | 81.59 (10.27) | 80.56 (14.77) | 79.70 (10.81) |
| **Height** † | (m) | mean (SD) | 1.72 (0.10) | 1.73 (0.09) | 1.73 (0.09) | 1.75 (0.08) | 1.73 (0.10) | 1.74 (0.09) |

* including the two S+OD subjects who were not matched to HR subjects.

† 1 subject (S+OD) had a missing body weight and height value.

Smokers were defined as light (<10 cigarettes or <5 cigars/pipes per day), moderate (10-19 cigarettes or 5-9 cigars/pipes per day), or heavy smokers (≥20 cigarettes or ≥10 cigars/pipes per day).

Current moderate or heavy alcohol use and moderate or heavy smoking were exclusion criteria for the HR group.

Abbreviations: SD, standard deviation.

#### Table S3. Serum vitamin D concentration group comparison adjusted for monthly estimated UVB dose using linear mixed model and AN(C)OVA.

|  |  |  | **Linear mixed models** | | | | | | |
| --- | --- | --- | --- | --- | --- | --- | --- | --- | --- |
|  |  |  |  | **S-OD** | **HR matched to S-OD** | **S+OD** | **HR matched to S+OD** | **Total S** | **Total HR** |
| **Parameter** | **Covariate** | **Model** |  | *N = 49* | *N = 49* | *N = 34* | *N = 34* | *N = 83* | *N = 83* |
| **Total 25-OH vitamin D** (nmol/L) | **Estimated monthly**  **UVB dose** | **Unadjusted analysis** | estimated marginal means (SE) | 42.5 (3.6) | 63.2 (3.4) | 46.0 (3.4) | 57.5 (3.8) | 43.9 (2.5) | 60.9 (2.5) |
|  |  |  | *p* value | **<.001** | | **0.029** | | **<.001** | |
|  |  | **Adjusted analysis** | estimated marginal means (SE) | 44.6 (3.5) | 61.2 (3.3) | 45.8 (3.4) | 57.7 (3.8) | 44.4 (2.5) | 60.4 (2.5) |
|  |  |  | *p* value | **0.001** | | **0.025** | | **<.001** | |
|  |  | **Adjusted analysis, including interaction term** | estimated marginal means (SE) | 44.7 (3.7) | 61.3 (3.4) | 45.9 (3.4) | 57.8 (3.8) | 44.4 (2.5) | 60.4 (2.5) |
|  |  |  | *p* value (p-value interaction term) | **0.002** (0.843) | | **0.026** (0.867) | | **<.001** (0.848) | |
|  |  |  |  |  |  |  |  |  |  |
|  |  |  | **AN(C)OVA** | | |  |  |  |  |
|  |  |  |  | **S-OD** | **S+OD *** |  |  |  |  |
| **Parameter** | **Covariate** | **Model** |  | *N = 49* | *N = 36* |  |  |  |  |
| **Total 25-OH vitamin D** (nmol/L) | **Estimated monthly**  **UVB dose** | **Unadjusted analysis** | estimated marginal means (SE) | 42.5 (3.3) | 45.4 (3.8) |  |  |  |  |
|  |  |  | *p* value | 0.569 | |  |  |  |  |
|  |  | **Adjusted analysis** | estimated marginal means (SE) | 42.6 (3.3) | 45.2 (3.8) |  |  |  |  |
|  |  |  | *p* value | 0.604 | |  |  |  |  |
|  |  | **Adjusted analysis, including interaction term** | estimated marginal means (SE) | 42.8 (3.3) | 45.3 (3.8) |  |  |  |  |
|  |  |  | *p* value (*p* value interaction term) | 0.603 (0.137) | |  |  |  |  |

Linear mixed models were performed for (adjusted) matched paired comparisons (stroke *vs.* HR groups), while AN(C)OVA was performed for adjusted unpaired comparisons (S-OD *vs.* S+OD). Dependent variable and covariate for the adjusted comparisons were: serum vitamin D adjusted for estimated monthly UVB dose in Germany (derived from reference 20; O’Neill CM, et al. Nutrients. (2016) 8:533). The interaction between parameter and covariate was also assessed with models including an additional covariate x study group interaction term. An interaction term was considered significant at *p* value < 0.10.

* including the two S+OD subjects who were not matched to a HR subject.

1 subject (S-OD) had a missing value for vitamin D.

bold *p* values are <0.05.

Abbreviations: SE, standard error; UVB, ultraviolet B.

#### Table S4. Fasted blood concentration of vitamin B6 and B12, folate, and homocysteine when excluding metformin users.

|  |  |  | ***S-OD*** | ***HR matched to S-OD*** | ***S+OD*** | ***HR matched to S+OD*** | ***Total S*** | ***Total HR*** | ***S+OD **** |
| --- | --- | --- | --- | --- | --- | --- | --- | --- | --- |
|  |  |  | *N = 40* | *N = 40* | *N = 32* | *N = 32* | *N = 72* | *N = 72* | *N = 34* |
| **Vitamin B6** | (nmol/L) | mean (SD) | 118.0 (268.8) | 103.1 (28.7) | 76.5 (22.5) | 116.3 (52.2) | 99.0 (198.7) | 109.0 (41.1) | 76.2 (22.1) |
|  |  | median (Q1-Q3) | 67.0 (57.0-81.0) | 95.5 (81.5-114.5) | 74.0 (62.0-92.0) | 99.5 (83.0-136.5) | 67.5 (58.0-83.5) | 96.0 (82.5-122.5) | 74.0 (62.0-84.0) |
|  |  | *p value* | **<.001 §** | | **<.001 §** | | **<.001 §** | | 0.304 ** |
| **Vitamin B12** | (pmol/L) | mean (SD) | 320.7 (118.3) | 272.5 (82.8) | 359.8 (155.7) | 279.4 (120.8) | 338.3 (136.8) | 275.6 (100.7) | 353.8 (154.5) |
|  |  | median (Q1-Q3) | 297.0 (223.0-383.0) | 275.5 (214.0-326.0) | 314.0 (247.5-433.5) | 279.0 (191.5-331.5) | 309.0 (244.0-399.0) | 277.5 (209.5-331.5) | 314.0 (246.0-420.0) |
|  |  | *p value* | 0.188 § | | **0.018 §** | | **0.006 §** | | 0.469 ** |
| **Folic acid †** | (nmol/L) | mean (SD) | 18.1 (11.3) | 18.6 (10.5) | 17.2 (9.5) | 19.5 (10.7) | 17.7 (10.5) | 19.0 (10.5) | 18.9 (11.4) |
|  |  | median (Q1-Q3) | 13.3 (10.7-23.1) | 14.7 (11.4-23.9) | 14.6 (10.0-20.4) | 15.6 (12.9-25.1) | 13.8 (10.5-21.2) | 15.3 (11.8-24.5) | 15.3 (10.5-23.7) |
|  |  | *p value* | 0.445 § | | 0.383 § | | 0.217 § | | **0.792 **** |
| **Homocysteine** | (μmol/L) | mean (SD) | 14.8 (5.8) | 13.3 (3.8) | 12.9 (4.0) | 14.3 (6.1) | 14.0 (5.1) | 13.7 (4.9) | 12.5 (4.2) |
|  |  | median (Q1-Q3) | 13.7 (11.0-17.2) | 12.3 (10.4-15.0) | 12.8 (9.8-15.2) | 14.0 (10.0-17.1) | 13.4 (10.8-16.6) | 12.9 (10.0-15.7) | 12.1 (9.4-15.1) |
|  |  | *p value* | 0.342 § | | 0.358 § | | 0.947 § | | 0.082 ** |

missing values (e.g. due to lack of volume) ranged between 1 and a maximum of 4 cases per parameter within all 146 subjects.

* including the two S+OD subjects who were not matched to HR subjects.

† 5 subjects (2 S-OD, 1 HR matched to S-OD, and 2 HR matched to S+OD) had serum folate concentration above the upper limit of detection. In these cases, the upper limit of detection value was taken.

§ *p* value derived from a Wilcoxon signed rank test.

** *p* value derived from a Wilcoxon rank sum test using Monte Carlo simulation.

bold *p* values are <0.05

Abbreviations: Q1, Quartile 1; Q3, Quartile 3; SD, standard deviation.

#### Table S5. Correction for multiple testing on all blood vitamin and minerals levels per group comparison.

|  |  | ***S-OD vs. HR matched to S-OD*** | | ***S+OD vs. HR matched to S+OD*** | | ***Total S vs. Total HR*** | | ***S-OD vs. S+OD **** | |
| --- | --- | --- | --- | --- | --- | --- | --- | --- | --- |
|  |  | *original p value* | *Benjamini-Hochberg corrected p value* | *original p value* | *Benjamini-Hochberg corrected p value* | *original p value* | *Benjamini-Hochberg corrected p value* | *original p value* | *Benjamini-Hochberg corrected p value* |
| **Vitamin B1** | (nmol/L) | **0.041 §** | ***0.041*** | **0.012 ‡** | ***0.012*** | **0.009 ‡** | ***0.009*** | 0.697 ** | *0.767* |
| **Vitamin B2** | (nmol/L) | **<.001 ‡** | ***0.001*** | **<.001 ‡** | ***0.002*** | **<.001 ‡** | ***0.001*** | 0.556 ** | *0.767* |
| **Vitamin B6** | (nmol/L) | **<.001 §** | ***0.001*** | **<.001 §** | ***0.002*** | **<.001 §** | ***0.001*** | 0.325 ** | *0.767* |
| **Vitamin B12** | (pmol/L) | 0.062 ‡ | *0.062* | **0.012 §** | ***0.012*** | **0.003 ‡** | ***0.003*** | 0.342 ** | *0.767* |
| **Folic acid** | (nmol/L) | 0.145 § | *0.145* | 0.282 § | *0.282* | 0.064 § | *0.064* | 0.999 ** | *0.999* |
| **Vitamin A** | (μmol/L) | **0.006 ‡** | ***0.006*** | **0.008 ‡** | ***0.01*** | **<.001 ‡** | ***0.001*** | 0.086 ǁ | *0.767* |
| **Total 25-OH vit D** | (nmol/L) | **<.001 ‡** | ***0.001*** | **0.022 §** | ***0.022*** | **<.001 ‡** | ***0.001*** | 0.569 ǁ | *0.767* |
| **Vitamin E** | (μmol/L) | **<.001 ‡** | ***0.001*** | **<.001 §** | ***0.002*** | **<.001 §** | ***0.001*** | 0.468 ǁ | *0.767* |
| **Selenium** | (μmol/L) | **<.001 ‡** | ***0.001*** | **0.018 ‡** | ***0.018*** | **<.001 ‡** | ***0.001*** | 0.358 ǁ | *0.767* |
| **Magnesium** | (mmol/L) | 0.352 ‡ | *0.352* | 0.477 ‡ | *0.477* | 0.478 § | *0.478* | 0.664 ** | *0.767* |
| **Zinc** | (μmol/L) | **0.025 §** | ***0.025*** | 0.335 § | *0.335* | 0.310 § | *0.310* | 0.454 ** | *0.767* |

Because of the exploratory nature of the study, no correction for multiple testing was applied, except for this example of a domain-specific Benjamini-Hochberg correction on all blood vitamin and minerals levels per group comparison.

* including the two S+OD subjects who were not matched to a HR subject.

‡ *p* value derived from a paired-t-test.

§ *p* value derived from a Wilcoxon signed rank test.

ǁ *p* value derived from two sample t-test with the Pooled method (equal variances).

** *p* value derived from a Wilcoxon rank sum test using Monte Carlo simulation.

bold *p* values are <0.05.

#### Table S6. Serum glucose concentration group comparison adjusted for medical history of diabetes dose using linear mixed models.

| **Linear mixed models** | | | | | |
| --- | --- | --- | --- | --- | --- |
|  |  |  |  | **Total S** | **Total HR** |
| **Parameter** | **Covariate** | **Model** |  | *N = 83* | *N = 83* |
| **Glucose** (mmol/L) | **Medical history of diabetes (yes/no)** | **Unadjusted analysis** | estimated marginal means (SE) | 6.32 (0.23) | 5.50 (0.06) |
|  |  |  | *p value* | **0.001** | |
|  |  | **Adjusted analysis** | estimated marginal means (SE) | 5.93 (0.19) | 5.89 (0.09) |
|  |  |  | *p value* | 0.858 | |

Linear mixed models were performed for adjusted matched paired comparisons (Total S *vs.* total HR only). Dependent variable and covariate for the adjusted comparisons were: serum glucose adjusted for medical history of diabetes. The interaction between parameter and covariate was not assessed since none of the HR subjects had a medical history of diabetes.

1 subject (S-OD) had a missing value for glucose.

bold *p* values are <0.05.

Abbreviations: SE, standard error.

#### Table S7. Plasma coenzyme Q10, serum cholesterol, and serum vitamin D concentration group comparison adjusted for use of HMG CoA reductase inhibitors (statins) using linear mixed models.

| **Linear mixed models** | | | | | |
| --- | --- | --- | --- | --- | --- |
|  |  |  |  | **Total S** | **Total HR** |
| **Parameter** | **Covariate** | **Model** |  | *N = 83* | *N = 83* |
| **Coenzyme Q10** (μmol/L) | **Use of HMG CoA reductase inhibitors (yes/no)** | **Unadjusted analysis** | estimated marginal means (SE) | 0.712 (0.038) | 1.531 (0.056) |
|  |  |  | *p value* | **<.001** | |
|  |  | **Adjusted analysis** | estimated marginal means (SE) | 0.887 (0.053) | 1.359 (0.068) |
|  |  |  | *p value* | **<.001** | |
| **Total cholesterol** (mmol/L) | **Use of HMG CoA reductase inhibitors (yes/no)** | **Unadjusted analysis** | estimated marginal means (SE) | 3.48 (0.10) | 5.55 (0.09) |
|  |  |  | *p value* | **<.001** | |
|  |  | **Adjusted analysis** | estimated marginal means (SE) | 4.07 (0.12) | 4.97 (0.13) |
|  |  |  | *p value* | **<.001** | |
| **Total 25-OH vitamin D** (nmol/L) | **Use of HMG CoA reductase inhibitors (yes/no)** | **Unadjusted analysis** | estimated marginal means (SE) | 43.9 (2.5) | 60.9 (2.5) |
|  |  |  | *p value* | **<.001** | |
|  |  | **Adjusted analysis** | estimated marginal means (SE) | 45.1 (3.9) | 59.7 (3.8) |
|  |  |  | *p value* | **0.037** | |

Linear mixed models were performed for adjusted matched paired comparisons (Total S *vs.* total HR only). Dependent variables and covariate for the adjusted comparisons were: serum cholesterol, serum vitamin D, and plasma coQ10 adjusted for use of HMG CoA reductase inhibitors. The interaction between parameters and covariate was not assessed since none of the HR subjects had record of HMG CoA reductase inhibitors use.

2 subjects (1 S-OD and 1 S+OD) had a missing value for coenzyme Q10, and 1 subject (S-OD) had missing values for cholesterol and vitamin D.

bold *p* values are <0.05.

Abbreviations: SE, standard error. HMG CoA, 3-hydroxy-3-methylglutaryl coenzyme A.

#### Table S8. 1-day food diary: reported energy and macronutrient intake.

|  |  |  | ***Total S*** | ***Total HR*** | *Total S vs.* |
| --- | --- | --- | --- | --- | --- |
|  | |  | *N = 83* | *N = 83* | *Total HR* |
| **Intake per day** | |  |  | | *p value* |
| **Energy** | (kcal) | mean (SD) | 1857.2 (550.1) | 2168.5 (597.0) | **<.001 ‡** |
|  |  | median (Q1-Q3) | 1861.9 (1442.1-2278.4) | 2119.5 (1826.5-2444.3) |  |
|  | (kcal/kg body weight) | mean (SD) | 23.4 (7.5) | 27.5 (7.7) | **<.001 ‡** |
|  |  | median (Q1-Q3) | 23.1 (17.9-27.7) | 27.8 (21.3-32.1) |  |
| **Fat** | (g) | mean (SD) | 80.8 (31.3) | 94.4 (35.2) | **0.005 ‡** |
|  |  | median (Q1-Q3) | 83.9 (53.7-102.6) | 95.1 (67.4-117.9) |  |
|  | (g/kg body weight) | mean (SD) | 1.01 (0.41) | 1.20 (0.46) | **0.004 ‡** |
|  |  | median (Q1-Q3) | 1.05 (0.71-1.26) | 1.19 (0.84-1.50) |  |
|  | (En%) | mean (SD) | 38.8 (8.6) | 39.3 (10.3) | 0.707 ‡ |
|  |  | median (Q1-Q3) | 40.0 (32.5-43.3) | 39.6 (31.8-46.4) |  |
| **Protein** | (g) | mean (SD) | 73.4 (22.2) | 89.2 (28.9) | **<.001 ‡** |
|  |  | median (Q1-Q3) | 72.5 (58.1-87.0) | 91.9 (69.5-102.4) |  |
|  | (g/kg body weight) | mean (SD) | 0.93 (0.33) | 1.14 (0.39) | **<.001 ‡** |
|  |  | median (Q1-Q3) | 0.84 (0.74-1.14) | 1.17 (0.84-1.38) |  |
|  | (En%) | mean (SD) | 16.1 (3.2) | 16.9 (5.2) | 0.682 § |
|  |  | median (Q1-Q3) | 15.7 (13.7-18.6) | 15.5 (13.4-19.4) |  |
| **Carbohydrates** | (g) | mean (SD) | 198.5 (66.8) | 222.8 (89.4) | **0.036 ‡** |
|  |  | median (Q1-Q3) | 198.3 (154.9-233.0) | 209.8 (155.8-274.9) |  |
|  | (g/kg body weight) | mean (SD) | 2.50 (0.89) | 2.82 (1.08) | **0.048 ‡** |
|  |  | median (Q1-Q3) | 2.51 (1.94-2.89) | 2.84 (2.06-3.49) |  |
|  | Carbohydrates (En%) | mean (SD) | 42.8 (8.4) | 41.0 (11.5) | 0.183 ‡ |
|  |  | median (Q1-Q3) | 43.1 (37.7-47.6) | 40.1 (33.2-48.4) |  |
| **Fiber** | (g) | mean (SD) | 18.5 (6.7) | 21.6 (9.5) | **0.029 §** |
|  |  | median (Q1-Q3) | 17.4 (14.9-22.4) | 20.3 (15.9-26.8) |  |
|  | (g/kg body weight) | mean (SD) | 0.24 (0.10) | 0.27 (0.12) | **0.011 ‡** |
|  |  | median (Q1-Q3) | 0.22 (0.17-0.28) | 0.26 (0.19-0.35) |  |
|  | (En%) | mean (SD) | 2.0 (0.6) | 2.1 (0.9) | 0.821 § |
|  |  | median (Q1-Q3) | 2.0 (1.6-2.3) | 1.8 (1.5-2.6) |  |
| **Water** | (g) | mean (SD) | 2332.7 (877.4) | 2783.9 (1272.4) | **0.001 §** |
|  |  | median (Q1-Q3) | 2201.8 (1768.3-2878.9) | 2692.5 (2094.9-3203.8) |  |
|  | (g/kg body weight) | mean (SD) | 29.55 (10.96) | 35.69 (17.72) | **0.004 §** |
|  |  | median (Q1-Q3) | 28.43 (22.12-36.88) | 32.69 (26.18-43.85) |  |

Table S10. 1-day food diary: reported energy and macronutrient intake (continued).

|  |  |  | ***Total S*** | ***Total HR*** | *Total S vs.* |
| --- | --- | --- | --- | --- | --- |
|  | |  | *N = 83* | *N = 83* | *Total HR* |
| **Intake per day** | |  |  | | *p value* |
| **Vitamin A** | (μg) | mean (SD) | 1558.5 (1271.1) | 1205.1 (1443.8) | **0.020 §** |
|  |  | median (Q1-Q3) | 1015.8 (713.6-2473.9) | 808.3 (567.7-1384.7) |  |
| **Vitamin B1** | (mg) | mean (SD) | 1.01 (0.47) | 1.20 (0.58) | 0.055 § |
|  |  | median (Q1-Q3) | 1.00 (0.70-1.20) | 1.10 (0.80-1.40) |  |
| **Vitamin B2** | (mg) | mean (SD) | 1.15 (0.56) | 1.66 (0.87) | **<.001 §** |
|  |  | median (Q1-Q3) | 1.10 (0.80-1.50) | 1.40 (1.10-1.90) |  |
| **Vitamin B6** | (mg) | mean (SD) | 1.17 (0.58) | 1.72 (0.71) | **<.001 §** |
|  |  | median (Q1-Q3) | 1.10 (0.70-1.40) | 1.70 (1.30-2.10) |  |
| **Folate** | (μg) | mean (SD) | 197.4 (82.3) | 268.6 (115.1) | **<.001 ‡** |
|  |  | median (Q1-Q3) | 185.8 (141.7-230.3) | 256.8 (178.5-323.6) |  |
| **Vitamin B12** | (μg) | mean (SD) | 5.43 (3.26) | 6.38 (8.26) | 0.754 § |
|  |  | median (Q1-Q3) | 4.35 (3.10-7.30) | 5.00 (3.20-7.30) |  |
| **Vitamin C** | (mg) | mean (SD) | 101.2 (71.9) | 114.6 (74.4) | 0.188 ‡ |
|  |  | median (Q1-Q3) | 90.3 (43.4-131.6) | 97.4 (62.8-155.4) |  |
| **Vitamin D** | (μg) | mean (SD) | 1.69 (1.22) | 4.85 (7.99) | **<.001 §** |
|  |  | median (Q1-Q3) | 1.40 (0.90-2.20) | 3.00 (1.20-4.70) |  |
| **Vitamin E (Aq)** | (mg) | mean (SD) | 7.20 (4.38) | 11.53 (6.52) | **<.001 ‡** |
|  |  | median (Q1-Q3) | 5.80 (4.10-9.50) | 10.20 (6.70-15.10) |  |
| **Vitamin E** | (mg) | mean (SD) | 6.99 (4.36) | 10.60 (6.05) | **<.001 ‡** |
|  |  | median (Q1-Q3) | 5.70 (3.90-9.50) | 9.20 (6.10-13.80) |  |
| **Calcium** | (mg) | mean (SD) | 943.7 (446.8) | 942.3 (494.1) | 0.993 ‡ |
|  |  | median (Q1-Q3) | 903.9 (628.2-1152.2) | 879.1 (541.6-1306.7) |  |
| **Magnesium** | (mg) | mean (SD) | 269.7 (91.3) | 384.8 (130.1) | **<.001 ‡** |
|  |  | median (Q1-Q3) | 256.7 (208.2-306.2) | 367.2 (279.4-433.9) |  |
| **Iron** | (mg) | mean (SD) | 9.47 (3.50) | 13.63 (4.96) | **<.001 §** |
|  |  | median (Q1-Q3) | 9.00 (7.10-11.30) | 13.40 (10.40-15.30) |  |
| **Zinc** | (mg) | mean (SD) | 11.0 (4.3) | 11.6 (4.4) | 0.364 ‡ |
|  |  | median (Q1-Q3) | 10.2 (8.4-14.1) | 11.1 (8.4-13.8) |  |
| **Cholesterol** | (mg) | mean (SD) | 293.2 (146.1) | 416.6 (304.2) | **0.003 §** |
|  |  | median (Q1-Q3) | 279.3 (187.9-372.4) | 371.0 (210.4-543.6) |  |
| **Carotene** | (mg) | mean (SD) | 3.26 (4.20) | 3.57 (7.81) | 0.693 § |
|  |  | median (Q1-Q3) | 1.50 (0.90-3.50) | 1.60 (0.90-3.30) |  |
| **Sodium** | (mg) | mean (SD) | 2770.6 (988.5) | 2499.8 (932.9) | **0.035 ‡** |
|  |  | median (Q1-Q3) | 2637.1 (2152.0-3210.1) | 2499.9 (1733.4-3146.9) |  |
| **Potassium** | (mg) | mean (SD) | 2431.1 (747.0) | 3417.6 (1125.6) | **<.001 §** |
|  |  | median (Q1-Q3) | 2416.6 (1994.0-2962.1) | 3364.9 (2666.2-4049.3) |  |
| **Phosphor** | (mg) | mean (SD) | 1211.2 (410.7) | 1462.5 (538.1) | **<.001 ‡** |
|  |  | median (Q1-Q3) | 1161.3 (981.4-1418.2) | 1332.7 (1085.3-1780.8) |  |

Table S10. 1-day food diary: reported energy and macronutrient intake (continued).

|  |  |  | ***Total S*** | ***Total HR*** | *Total S vs.* |
| --- | --- | --- | --- | --- | --- |
|  | |  | *N = 83* | *N = 83* | *Total HR* |
| **Intake per day** | |  |  | | *p value* |
| **DHA** | (g) | mean (SD) | 0.08 (0.26) | 0.32 (0.59) | not available |
|  |  | median (Q1-Q3) | 0.00 (0.00-0.00) | 0.10 (0.00-0.30) |  |
| **EPA** | (g) | mean (SD) | 0.03 (0.14) | 0.19 (0.51) | not available |
|  |  | median (Q1-Q3) | 0.00 (0.00-0.00) | 0.00 (0.00-0.10) |  |
| **ALA** | (g) | mean (SD) | 0.99 (0.58) | 1.97 (3.60) | not available |
|  |  | median (Q1-Q3) | 0.90 (0.60-1.20) | 1.20 (0.80-1.90) |  |

1 subject (Total S) had missing records on all nutritional intake parameters because of not completing the diary and 1 subject (Total S) had a missing body weight record and therefore missing records on parameters expressed per kg body weight.

‡ *p* value derived from a paired-t-test.

§ *p* value derived from a Wilcoxon signed rank test.

bold *p* values are <0.05.

Abbreviations: ALA, alpha-linolenic acid; DHA, docosahexaenoic acid; EPA, eicosapentaenoic acid; SD, standard deviation; Q1, Quartile 1; Q3, Quartile 3.

#### Table S9. EQ-5D-5L scores on separate categories.

|  |  |  |  | ***S-OD*** | ***HR matched to S-OD*** | ***S+OD*** | ***HR matched to S+OD*** | ***Total S*** | ***Total HR*** | ***S+OD **** |
| --- | --- | --- | --- | --- | --- | --- | --- | --- | --- | --- |
|  |  |  |  | *N = 49* | *N = 49* | *N = 34* | *N = 34* | *N = 83* | *N = 83* | *N = 36* |
| **EQ-5D-5L** | **Mobility** | No problems (Level 1) | n (%) | 26 (53.1) | 48 (98.0) | 6 (17.6) | 33 (97.1) | 32 (38.6) | 81 (97.6) | 6 (16.7) |
|  |  | Slight problems (Level 2) |  | 8 (16.3) | 0 (0.0) | 3 (8.8) | 1 (2.9) | 11 (13.3) | 1 (1.2) | 4 (11.1) |
|  |  | Moderate problems (Level 3) |  | 5 (10.2) | 1 (2.0) | 3 (8.8) | 0 (0.0) | 8 (9.6) | 1 (1.2) | 3 (8.3) |
|  |  | Severe problems (Level 4) |  | 3 (6.1) | 0 (0.0) | 4 (11.8) | 0 (0.0) | 7 (8.4) | 0 (0.0) | 5 (13.9) |
|  |  | Extreme problems (Level 5) |  | 7 (14.3) | 0 (0.0) | 18 (52.9) | 0 (0.0) | 25 (30.1) | 0 (0.0) | 18 (50.0) |
|  | **Self-Care** | No problems (Level 1) | n (%) | 36 (73.5) | 49 (100.0) | 7 (20.6) | 34 (100.0) | 43 (51.8) | 83 (100.0) | 8 (22.2) |
|  |  | Slight problems (Level 2) |  | 3 (6.1) | 0 (0.0) | 3 (8.8) | 0 (0.0) | 6 (7.2) | 0 (0.0) | 3 (8.3) |
|  |  | Moderate problems (Level 3) |  | 5 (10.2) | 0 (0.0) | 9 (26.5) | 0 (0.0) | 14 (16.9) | 0 (0.0) | 9 (25.0) |
|  |  | Severe problems (Level 4) |  | 4 (8.2) | 0 (0.0) | 9 (26.5) | 0 (0.0) | 13 (15.7) | 0 (0.0) | 10 (27.8) |
|  |  | Extreme problems (Level 5) |  | 1 (2.0) | 0 (0.0) | 6 (17.6) | 0 (0.0) | 7 (8.4) | 0 (0.0) | 6 (16.7) |
|  | **Usual Activities** | No problems (Level 1) | n (%) | 23 (46.9) | 48 (98.0) | 3 (8.8) | 33 (97.1) | 26 (31.3) | 81 (97.6) | 4 (11.1) |
|  |  | Slight problems (Level 2) |  | 8 (16.3) | 0 (0.0) | 2 (5.9) | 1 (2.9) | 10 (12.0) | 1 (1.2) | 2 (5.6) |
|  |  | Moderate problems (Level 3) |  | 8 (16.3) | 1 (2.0) | 7 (20.6) | 0 (0.0) | 15 (18.1) | 1 (1.2) | 7 (19.4) |
|  |  | Severe problems (Level 4) |  | 5 (10.2) | 0 (0.0) | 9 (26.5) | 0 (0.0) | 14 (16.9) | 0 (0.0) | 9 (25.0) |
|  |  | Extreme problems (Level 5) |  | 5 (10.2) | 0 (0.0) | 13 (38.2) | 0 (0.0) | 18 (21.7) | 0 (0.0) | 14 (38.9) |
|  | **Pain / Discomfort** | No problems (Level 1) | n (%) | 36 (73.5) | 41 (83.7) | 23 (67.6) | 26 (76.5) | 59 (71.1) | 67 (80.7) | 24 (66.7) |
|  |  | Slight problems (Level 2) |  | 4 (8.2) | 6 (12.2) | 2 (5.9) | 7 (20.6) | 6 (7.2) | 13 (15.7) | 2 (5.6) |
|  |  | Moderate problems (Level 3) |  | 6 (12.2) | 2 (4.1) | 5 (14.7) | 1 (2.9) | 11 (13.3) | 3 (3.6) | 6 (16.7) |
|  |  | Severe problems (Level 4) |  | 3 (6.1) | 0 (0.0) | 2 (5.9) | 0 (0.0) | 5 (6.0) | 0 (0.0) | 2 (5.6) |
|  |  | Extreme problems (Level 5) |  | 0 (0.0) | 0 (0.0) | 2 (5.9) | 0 (0.0) | 2 (2.4) | 0 (0.0) | 2 (5.6) |
|  | **Anxiety / Depression †** | No problems (Level 1) | n (%) | 34 (69.4) | 49 (100.0) | 19 (55.9) | 34 (100.0) | 53 (63.9) | 83 (100.0) | 21 (58.3) |
|  |  | Slight problems (Level 2) |  | 14 (28.6) | 0 (0.0) | 6 (17.6) | 0 (0.0) | 20 (24.1) | 0 (0.0) | 6 (16.7) |
|  |  | Moderate problems (Level 3) |  | 1 (2.0) | 0 (0.0) | 4 (11.8) | 0 (0.0) | 5 (6.0) | 0 (0.0) | 4 (11.1) |
|  |  | Severe problems (Level 4) |  | 0 (0.0) | 0 (0.0) | 2 (5.9) | 0 (0.0) | 2 (2.4) | 0 (0.0) | 2 (5.6) |
|  |  | Extreme problems (Level 5) |  | 0 (0.0) | 0 (0.0) | 1 (2.9) | 0 (0.0) | 1 (1.2) | 0 (0.0) | 1 (2.8) |

* including the two S+OD subjects who were not matched to HR subjects.

† 2 subjects (S+OD) had missing record on EQ-5D-5L Anxiety / Depression score.

Abbreviations: EQ-5D-5L, EuroQol 5-dimensions 5-level.

#### Table S10. Barthel scores on separate categories.

|  |  |  |  | ***S-OD*** | ***HR matched to S-OD*** | ***S+OD*** | ***HR matched to S+OD*** | ***Total S*** | ***Total HR*** | ***S+OD **** |
| --- | --- | --- | --- | --- | --- | --- | --- | --- | --- | --- |
|  |  |  |  | *N = 49* | *N = 49* | *N = 34* | *N = 34* | *N = 83* | *N = 83* | *N = 36* |
| **Barthel Score** | **Bowels** | Incontinent (score 0) | n (%) | 0 (0.0) | 0 (0.0) | 7 (20.6) | 0 (0.0) | 7 (8.4) | 0 (0.0) | 7 (19.4) |
|  |  | Occasional accident (score 5) |  | 0 (0.0) | 0 (0.0) | 7 (20.6) | 0 (0.0) | 7 (8.4) | 0 (0.0) | 7 (19.4) |
|  |  | Continent (score 10) |  | 49 (100.0) | 49 (100.0) | 20 (58.8) | 34 (100.0) | 69 (83.1) | 83 (100.0) | 22 (61.1) |
|  | **Bladder** | Incontinent (score 0) | n (%) | 1 (2.0) | 0 (0.0) | 8 (23.5) | 0 (0.0) | 9 (10.8) | 0 (0.0) | 8 (22.2) |
|  |  | Occasional accident (score 5) |  | 2 (4.1) | 0 (0.0) | 11 (32.4) | 0 (0.0) | 13 (15.7) | 0 (0.0) | 11 (30.6) |
|  |  | Continent (score 10) |  | 46 (93.9) | 49 (100.0) | 15 (44.1) | 34 (100.0) | 61 (73.5) | 83 (100.0) | 17 (47.2) |
|  | **Grooming** | Needs to help with personal care (score 0) | n (%) | 2 (4.1) | 0 (0.0) | 14 (41.2) | 0 (0.0) | 16 (19.3) | 0 (0.0) | 14 (38.9) |
|  |  | Independent face/hair/teeth/shaving (score 5) |  | 47 (95.9) | 49 (100.0) | 20 (58.8) | 34 (100.0) | 67 (80.7) | 83 (100.0) | 22 (61.1) |
|  | **Toilet use** | Dependent (score 0) | n (%) | 0 (0.0) | 0 (0.0) | 9 (26.5) | 0 (0.0) | 9 (10.8) | 0 (0.0) | 9 (25.0) |
|  |  | Needs some help (score 5) |  | 6 (12.2) | 0 (0.0) | 10 (29.4) | 0 (0.0) | 16 (19.3) | 0 (0.0) | 10 (27.8) |
|  |  | Independent (score 10) |  | 43 (87.8) | 49 (100.0) | 15 (44.1) | 34 (100.0) | 58 (69.9) | 83 (100.0) | 17 (47.2) |
|  | **Feeding** | Unable (score 0) | n (%) | 0 (0.0) | 0 (0.0) | 1 (2.9) | 0 (0.0) | 1 (1.2) | 0 (0.0) | 1 (2.8) |
|  |  | Needs help or requires modified diet (score 5) |  | 2 (4.1) | 0 (0.0) | 22 (64.7) | 0 (0.0) | 24 (28.9) | 0 (0.0) | 22 (61.1) |
|  |  | Independent (score 10) |  | 47 (95.9) | 49 (100.0) | 11 (32.4) | 34 (100.0) | 58 (69.9) | 83 (100.0) | 13 (36.1) |
|  | **Transfers** (bed to chair and back) | Unable, no sitting balance (score 0) | n (%) | 0 (0.0) | 0 (0.0) | 0 (0.0) | 0 (0.0) | 0 (0.0) | 0 (0.0) | 0 (0.0) |
|  |  | Major help, can sit (score 5) |  | 3 (6.1) | 0 (0.0) | 15 (44.1) | 0 (0.0) | 18 (21.7) | 0 (0.0) | 15 (41.7) |
|  |  | Minor help (score 10) |  | 3 (6.1) | 0 (0.0) | 10 (29.4) | 0 (0.0) | 13 (15.7) | 0 (0.0) | 11 (30.6) |
|  |  | Independent (score 15) |  | 43 (87.8) | 49 (100.0) | 9 (26.5) | 34 (100.0) | 52 (62.7) | 83 (100.0) | 10 (27.8) |
|  | **Mobility** (on level surfaces) | Immobile (score 0) | n (%) | 2 (4.1) | 0 (0.0) | 11 (32.4) | 0 (0.0) | 13 (15.7) | 0 (0.0) | 11 (30.6) |
|  |  | Wheelchair independent including corners (score 5) |  | 7 (14.3) | 0 (0.0) | 12 (35.3) | 0 (0.0) | 19 (22.9) | 0 (0.0) | 13 (36.1) |
|  |  | Walks with help of one person (score 10) |  | 10 (20.4) | 0 (0.0) | 4 (11.8) | 0 (0.0) | 14 (16.9) | 0 (0.0) | 4 (11.1) |
|  |  | Independent (score 15) |  | 30 (61.2) | 49 (100.0) | 7 (20.6) | 34 (100.0) | 37 (44.6) | 83 (100.0) | 8 (22.2) |
|  | **Dressing** | Dependent (score 0) | n (%) | 1 (2.0) | 0 (0.0) | 16 (47.1) | 0 (0.0) | 17 (20.5) | 0 (0.0) | 16 (44.4) |
|  |  | Needs help but can do about half unaided (score 5) |  | 9 (18.4) | 0 (0.0) | 10 (29.4) | 0 (0.0) | 19 (22.9) | 0 (0.0) | 11 (30.6) |
|  |  | Independent (score 10) |  | 39 (79.6) | 49 (100.0) | 8 (23.5) | 34 (100.0) | 47 (56.6) | 83 (100.0) | 9 (25.0) |
|  | **Stairs** | Unable (score 0) | n (%) | 16 (32.7) | 0 (0.0) | 26 (76.5) | 0 (0.0) | 42 (50.6) | 0 (0.0) | 27 (75.0) |
|  |  | Needs help (score 5) |  | 9 (18.4) | 0 (0.0) | 4 (11.8) | 0 (0.0) | 13 (15.7) | 0 (0.0) | 4 (11.1) |
|  |  | Independent (score 10) |  | 24 (49.0) | 49 (100.0) | 4 (11.8) | 34 (100.0) | 28 (33.7) | 83 (100.0) | 5 (13.9) |
|  | **Bathing** | Dependent (score 0) | n (%) | 20 (40.8) | 0 (0.0) | 30 (88.2) | 0 (0.0) | 50 (60.2) | 0 (0.0) | 31 (86.1) |
|  |  | Independent (score 5) |  | 29 (59.2) | 49 (100.0) | 4 (11.8) | 34 (100.0) | 33 (39.8) | 83 (100.0) | 5 (13.9) |

* including the two S+OD subjects who were not matched to HR subjects.

#### Table S11. Subgroup analysis based upon their MNA-SF score.

|  |  |  |  | ***Stroke patients with MNA-SF score 0-11 (malnourished or at risk of malnutrition)*** | ***HR subjects matched to Stroke patients with MNA-SF score 0-11*** |  | ***Stroke patients with MNA-SF score 12-14 (normal nourished)*** | ***HR subjects matched to Stroke patients with MNA-SF score 12-14*** |  |
| --- | --- | --- | --- | --- | --- | --- | --- | --- | --- |
|  |  |  |  | *N = 44* | *N = 44* | *p value for comparison* | *N = 36* | *N = 36* | *p value for comparison* |
|  | **Vitamin B1** | (nmol/L) | mean (SD) | 123.0 (27.6) | 143.2 (29.8) | **0.005** | 139.1 (39.9) | 145.4 (19.6) | 0.073 |
|  |  |  | median (Q1-Q3) | 124 (106-139) | 144 (121-163) |  | 134 (116-161) | 143 (135-158) |  |
|  | **Vitamin B2** | (nmol/L) | mean (SD) | 230.2 (39.8) | 271.8 (40.5) | **<.001** | 245.8 (49.2) | 279.8 (39.7) | **<.001** |
|  |  |  | median (Q1-Q3) | 228 (207-252) | 269 (245-299) |  | 238 (218-263) | 284 (254-300) |  |
|  | **Vitamin B6** | (nmol/L) | mean (SD) | 67.8 (19.1) | 108.3 (34.3) | **<.001** | 128.1 (279.2) | 109.4 (48.8) | **0.010** |
|  |  |  | median (Q1-Q3) | 65 (55-81) | 102 (84-125) |  | 75 (62-86) | 94 (80-118) |  |
|  | **Vitamin B12** | (pmol/L) | mean (SD) | 345.9 (157.5) | 257.8 (89.7) | **0.003** | 312.7 (95.4) | 288.6 (109.8) | 0.268 |
|  |  |  | median (Q1-Q3) | 308.0 (237.5-409.5) | 271.5 (191.5-313.0) |  | 312.0 (237.0-383.0) | 275.5 (217.5-350.0) |  |
|  | **Folic acid †** | (nmol/L) | mean (SD) | 16.2 (9.7) | 18.7 (9.5) | 0.067 | 18.3 (10.6) | 19.0 (10.6) | 0.525 |
|  |  |  | median (Q1-Q3) | 13.1 (10.4-18.2) | 15.5 (12.7-22.4) |  | 13.3 (10.7-23.1) | 16.6 (10.7-25.9) |  |
|  | **Vitamin A** | (μmol/L) | mean (SD) | 2.12 (0.61) | 2.50 (0.52) | **0.016** | 2.25 (0.56) | 2.60 (0.45) | **0.012** |
|  |  |  | median (Q1-Q3) | 2.19 (1.71-2.53) | 2.38 (2.13-2.80) |  | 2.19 (1.81-2.70) | 2.49 (2.33-2.88) |  |
|  | **Total 25-OH vit D** | (nmol/L) | mean (SD) | 40.2 (23.9) | 59.2 (22.4) | **<.001** | 47.5 (21.2) | 62.5 (22.4) | **0.021** |
|  |  |  | median (Q1-Q3) | 29.9 (20.8-50.5) | 56.5 (41.3-72.3) |  | 43.0 (34.6-68.0) | 60.2 (47.4-71.7) |  |
|  | **Vitamin E** | (μmol/L) | mean (SD) | 26.3 (5.9) | 40.6 (12.7) | **<.001** | 25.6 (7.9) | 38.7 (8.4) | **<.001** |
|  |  |  | median (Q1-Q3) | 26.2 (22.0-28.8) | 38.7 (33.5-44.0) |  | 22.0 (19.7-30.8) | 36.5 (33.9-42.5) |  |
|  | **Selenium** | (μmol/L) | mean (SD) | 0.87 (0.23) | 1.00 (0.22) | **0.008** | 0.91 (0.18) | 1.05 (0.25) | **0.002** |
|  |  |  | median (Q1-Q3) | 0.87 (0.76-0.97) | 0.98 (0.84-1.12) |  | 0.90 (0.84-0.99) | 1.08 (0.85-1.16) |  |
|  | **Magnesium** | (mmol/L) | mean (SD) | 0.82 (0.10) | 0.85 (0.06) | 0.072 | 0.87 (0.08) | 0.86 (0.05) | 0.224 |
|  |  |  | median (Q1-Q3) | 0.84 (0.79-0.90) | 0.86 (0.81-0.89) |  | 0.88 (0.84-0.91) | 0.86 (0.82-0.89) |  |
|  | **Zinc** | (μmol/L) | mean (SD) | 13.8 (2.9) | 13.5 (2.2) | 0.532 | 14.0 (1.8) | 13.3 (1.4) | 0.204 |
|  |  |  | median (Q1-Q3) | 14 (12-16) | 13 (12-15) |  | 14 (12-15) | 13 (12-14) |  |
|  | **Homocysteine** | (μmol/L) | mean (SD) | 14.3 (6.0) | 13.2 (3.8) | 0.881 | 14.6 (4.6) | 14.4 (5.9) | 0.732 |
|  |  |  | median (Q1-Q3) | 13.4 (10.3-16.7) | 13.3 (10.8-15.2) |  | 13.8 (11.9-17.0) | 12.3 (10.1-17.3) |  |
|  | **Free choline** | (μmol/L) | mean (SD) | 7.94 (2.18) | 9.44 (2.55) | **0.009** | 8.74 (2.23) | 9.55 (3.03) | 0.226 |
|  |  |  | median (Q1-Q3) | 7.68 (6.29-9.02) | 9.31 (7.65-11.15) |  | 8.62 (7.24-9.09) | 9.23 (7.76-10.55) |  |
|  | **Uridine** | (μmol/L) | mean (SD) | 3.88 (1.36) | 4.23 (1.38) | 0.147 | 4.19 (1.18) | 4.05 (1.03) | 0.695 |
|  |  |  | median (Q1-Q3) | 3.65 (2.90-4.57) | 4.00 (3.40-5.00) |  | 4.00 (3.50-4.80) | 4.05 (3.31-4.85) |  |
|  | **Glucose** | (mmol/L) | mean (SD) | 6.31 (2.10) | 5.53 (0.67) | **0.014** | 6.37 (2.07) | 5.44 (0.44) | 0.097 |
|  |  |  | median (Q1-Q3) | 5.6 (5.1-6.8) | 5.5 (5.1-5.8) |  | 5.6 (5.2-6.4) | 5.5 (5.1-5.7) |  |
|  | **Coenzyme Q10** | (μmol/L) | mean (SD) | 0.668 (0.312) | 1.543 (0.504) | **<.001** | 0.778 (0.390) | 1.496 (0.514) | **<.001** |
|  |  |  | median (Q1-Q3) | 0.605 (0.428-0.773) | 1.507 (1.222-1.889) |  | 0.681 (0.522-0.923) | 1.398 (1.148-1.825) |  |
|  | **Total cholesterol** | (mmol/L) | mean (SD) | 3.52 (0.88) | 5.56 (0.93) | **<.001** | 3.47 (0.93) | 5.52 (0.70) | **<.001** |
|  |  |  | median (Q1-Q3) | 3.42 (2.96-3.90) | 5.54 (4.80-6.26) |  | 3.31 (2.83-3.89) | 5.39 (5.23-5.95) |  |
|  | **Albumin** | (g/L) | mean (SD) | 35.4 (4.6) | 39.4 (2.7) | **<.001** | 37.5 (3.6) | 39.9 (2.8) | **0.003** |
|  |  |  | median (Q1-Q3) | 36.3 (33.5-37.9) | 39.3 (37.6-41.4) |  | 37.8 (36.0-40.0) | 39.8 (38.2-41.6) |  |
|  | **Pre-albumin** | (g/L) | mean (SD) | 0.229 (0.055) | 0.292 (0.045) | **<.001** | 0.257 (0.046) | 0.302 (0.049) | **<.001** |
|  |  |  | median (Q1-Q3) | 0.220 (0.200-0.262) | 0.284 (0.262-0.326) |  | 0.257 (0.218-0.284) | 0.298 (0.267-0.328) |  |
|  | **Transferrin** | (g/L) | mean (SD) | 2.20 (0.46) | 2.54 (0.41) | **<.001** | 2.27 (0.35) | 2.48 (0.30) | **0.012** |
|  |  |  | median (Q1-Q3) | 2.11 (1.80-2.52) | 2.50 (2.30-2.80) |  | 2.26 (2.08-2.50) | 2.45 (2.30-2.60) |  |
|  | **Total carnitine** | (μmol/L) | mean (SD) | 50.0 (15.6) | 51.2 (8.7) | 0.475 | 47.5 (9.6) | 51.2 (9.9) | 0.272 |
|  |  |  | median (Q1-Q3) | 50 (42-59) | 51 (46-58) |  | 50 (42-54) | 52 (45-59) |  |
|  | **Free carnitine** | (μmol/L) | mean (SD) | 38.2 (11.8) | 39.0 (6.5) | 0.606 | 37.2 (6.3) | 38.8 (8.4) | 0.445 |
|  |  |  | median (Q1-Q3) | 39 (32-44) | 40 (33-44) |  | 37 (32-41) | 40 (33-45) |  |
|  | **Acylcarnitine** | (μmol/L) | mean (SD) | 11.8 (5.5) | 12.2 (3.9) | 0.404 | 11.2 (3.9) | 12.4 (4.5) | 0.519 |
|  |  |  | median (Q1-Q3) | 11 (9-14) | 13 (10-15) |  | 11 (9-14) | 12 (10-15) |  |
|  | **Creatinine** | (μmol/L) | mean (SD) | 81.5 (40.3) | 83.7 (14.8) | 0.055 | 84.6 (20.9) | 83.5 (14.6) | 0.570 |
|  |  |  | median (Q1-Q3) | 74 (65-92) | 83 (76-92) |  | 83 (72-94) | 84 (74-98) |  |
|  | **Creatine** | (μmol/L) | mean (SD) | 33.1 (20.7) | 34.9 (21.0) | 0.417 | 36.3 (29.6) | 32.1 (18.1) | 0.841 |
|  |  |  | median (Q1-Q3) | 26.5 (19.7-35.4) | 26.9 (18.3-48.7) |  | 28.7 (18.7-41.5) | 25.3 (17.7-40.1) |  |
|  | **C-reactive protein †** | (mg/L) | mean (SD) | 10.69 (32.00) | 1.87 (2.11) | **<.001** | 5.12 (7.86) | 1.76 (1.86) | **0.003** |
|  |  |  | median (Q1-Q3) | 2.90 (0.90-7.14) | 1.20 (0.85-1.90) |  | 2.09 (0.99-5.43) | 1.00 (0.50-2.30) |  |
|  | **Sodium** | (mmol/L) | mean (SD) | 139.89 (2.73) | 139.16 (1.99) | 0.090 | 140.56 (2.40) | 139.28 (1.61) | **0.008** |
|  |  |  | median (Q1-Q3) | 140.6 (138.7-142.0) | 138.9 (137.7-140.5) |  | 140.8 (139.3-142.0) | 139.3 (138.5-140.0) |  |
|  | **Osmolality** | (mOsm/kg) | mean (SD) | 293.94 (6.40) | 293.52 (6.26) | 0.849 | 296.43 (6.80) | 292.75 (4.95) | **<.001** |
|  |  |  | median (Q1-Q3) | 293.8 (291.5-297.0) | 293.0 (289.5-298.0) |  | 296.0 (293.0-299.0) | 291.0 (289.5-297.0) |  |
| **Plasma fatty acids** (mg/L) | **C18:2 n-6 (LA)** |  | mean (SD) | 685.1 (202.6) | 1044.1 (242.5) | **<.001** | 655.5 (174.7) | 1063.6 (205.3) | **<.001** |
|  |  |  | median (Q1-Q3) | 666.0 (534.6-776.7) | 1028.2 (902.5-1178.7) |  | 603.3 (534.1-741.4) | 1048.1 (929.7-1191.9) |  |
|  | **C18:3 n-3 (ALA)** |  | mean (SD) | 10.56 (3.64) | 22.64 (26.17) | **<.001** | 12.14 (5.68) | 17.54 (10.23) | **0.008** |
|  |  |  | median (Q1-Q3) | 10.01 (8.06-12.24) | 16.80 (12.05-23.41) |  | 10.59 (8.68-13.19) | 14.31 (10.64-20.18) |  |
|  | **C20:5n-3 (EPA)** |  | mean (SD) | 13.95 (4.76) | 29.62 (16.98) | **<.001** | 15.75 (6.36) | 28.84 (17.78) | **<.001** |
|  |  |  | median (Q1-Q3) | 13.15 (10.47-17.31) | 24.66 (15.91-41.15) |  | 14.97 (11.95-18.44) | 25.23 (15.22-39.56) |  |
|  | **C22:6n-3 (DHA)** |  | mean (SD) | 42.49 (11.81) | 48.95 (22.69) | 0.175 | 44.15 (16.35) | 52.33 (20.86) | **0.024** |
|  |  |  | median (Q1-Q3) | 41.03 (34.19-48.95) | 42.06 (34.32-59.95) |  | 39.80 (34.51-53.40) | 50.86 (36.26-67.09) |  |
|  | **Total SFA** |  | mean (SD) | 1004.2 (219.7) | 1233.7 (511.4) | **0.006** | 938.2 (227.4) | 1106.4 (266.1) | **0.006** |
|  |  |  | median (Q1-Q3) | 934.2 (825.3-1143.3) | 1092.8 (981.8-1304.9) |  | 896.8 (790.7-1033.8) | 1079.6 (926.4-1241.1) |  |
|  | **Total MUFA** |  | mean (SD) | 959.4 (255.7) | 1103.7 (630.5) | 0.350 | 868.4 (266.5) | 942.3 (276.4) | 0.193 |
|  |  |  | median (Q1-Q3) | 920.6 (774.9-1092.1) | 916.5 (817.8-1095.0) |  | 835.1 (680.4-1089.7) | 908.3 (761.1-1014.4) |  |
|  | **Total PUFA** |  | mean (SD) | 1044.9 (249.9) | 1466.8 (302.7) | **<.001** | 1005.7 (216.9) | 1490.3 (271.7) | **<.001** |
|  |  |  | median (Q1-Q3) | 993.0 (859.1-1144.2) | 1484.1 (1273.2-1599.5) |  | 947.0 (864.6-1142.0) | 1485.5 (1338.5-1652.0) |  |
|  | **Total n3 PUFA** |  | mean (SD) | 78.8 (18.0) | 117.1 (52.9) | **<.001** | 83.8 (25.6) | 113.3 (45.2) | **<.001** |
|  |  |  | median (Q1-Q3) | 74.9 (66.1-90.5) | 107.0 (82.1-142.1) |  | 78.8 (71.3-95.7) | 105.0 (78.9-142.8) |  |
|  | **Total n6 PUFA** |  | mean (SD) | 963.6 (236.9) | 1346.2 (280.2) | **<.001** | 919.7 (202.2) | 1373.6 (245.5) | **<.001** |
|  |  |  | median (Q1-Q3) | 932.2 (782.2-1060.1) | 1335.7 (1191.4-1476.6) |  | 869.4 (794.0-1053.3) | 1369.9 (1257.1-1508.5) |  |
|  | **Total FA** |  | mean (SD) | 3025.0 (651.7) | 3826.2 (1393.3) | **<.001** | 2827.9 (654.4) | 3559.1 (744.1) | **<.001** |
|  |  |  | median (Q1-Q3) | 2803.8 (2498.0-3457.5) | 3448.2 (3161.4-4025.4) |  | 2690.0 (2354.1-3226.9) | 3409.3 (3064.1-4116.8) |  |
| **Erythrocyte fatty acids** (weight% of total fatty acids) | **C18:2 n-6 (LA)** |  | mean (SD) | 9.96 (1.91) | 14.59 (2.48) | **<.001** | 10.28 (2.02) | 14.81 (1.79) | **<.001** |
|  |  |  | median (Q1-Q3) | 9.68 (8.46-10.73) | 14.13 (12.70-16.03) |  | 10.18 (8.64-11.33) | 14.80 (13.31-15.46) |  |
|  | **C18:3 n-3 (ALA)** |  | mean (SD) | 0.09 (0.06) | 0.27 (0.26) | **<.001** | 0.12 (0.07) | 0.23 (0.13) | **<.001** |
|  |  |  | median (Q1-Q3) | 0.09 (0.07-0.12) | 0.24 (0.17-0.28) |  | 0.12 (0.09-0.16) | 0.20 (0.15-0.29) |  |
|  | **C20:5n-3 (EPA)** |  | mean (SD) | 0.55 (0.18) | 0.82 (0.38) | **<.001** | 0.62 (0.26) | 0.85 (0.41) | **<.001** |
|  |  |  | median (Q1-Q3) | 0.56 (0.42-0.64) | 0.77 (0.54-1.06) |  | 0.58 (0.44-0.74) | 0.79 (0.53-1.12) |  |
|  | **C22:6n-3 (DHA)** |  | mean (SD) | 2.95 (0.60) | 2.51 (0.64) | **<.001** | 3.15 (0.85) | 2.69 (0.70) | **0.037** |
|  |  |  | median (Q1-Q3) | 2.79 (2.51-3.33) | 2.45 (2.06-2.92) |  | 3.01 (2.55-3.70) | 2.72 (2.27-3.15) |  |
|  | **Total SFA** |  | mean (SD) | 44.3 (2.1) | 42.3 (1.8) | **<.001** | 44.1 (2.0) | 42.1 (2.3) | **<.001** |
|  |  |  | median (Q1-Q3) | 44.3 (43.1-45.6) | 42.3 (40.7-43.6) |  | 44.1 (43.0-44.5) | 41.9 (40.5-43.9) |  |
|  | **Total MUFA** |  | mean (SD) | 21.8 (1.6) | 21.7 (2.1) | 0.612 | 21.4 (1.5) | 21.0 (2.2) | 0.454 |
|  |  |  | median (Q1-Q3) | 21.8 (20.7-23.0) | 21.3 (20.2-22.5) |  | 21.3 (20.4-22.0) | 20.6 (19.5-21.9) |  |
|  | **Total PUFA** |  | mean (SD) | 30.8 (2.1) | 33.5 (2.4) | **<.001** | 31.5 (2.6) | 34.4 (1.7) | **<.001** |
|  |  |  | median (Q1-Q3) | 31.2 (29.0-32.3) | 32.9 (32.1-35.2) |  | 32.2 (29.9-33.4) | 34.7 (33.5-35.4) |  |
|  | **Total n3 PUFA** |  | mean (SD) | 5.0 (0.8) | 5.0 (1.2) | 0.827 | 5.4 (1.2) | 5.2 (1.2) | 0.646 |
|  |  |  | median (Q1-Q3) | 4.8 (4.5-5.5) | 4.9 (4.3-5.5) |  | 5.3 (4.8-6.1) | 5.1 (4.4-6.0) |  |
|  | **Total n6 PUFA** |  | mean (SD) | 25.8 (2.1) | 28.4 (2.8) | **<.001** | 26.0 (2.3) | 29.2 (1.8) | **<.001** |
|  |  |  | median (Q1-Q3) | 25.7 (24.1-27.3) | 28.1 (26.8-29.9) |  | 26.2 (24.8-27.5) | 29.2 (28.4-30.5) |  |
|  | **EQ-5D-5L Index Value** |  | mean (SD) | 0.55 (0.37) | 0.98 (0.04) | **<.001** | 0.78 (0.29) | 0.98 (0.05) | **<.001** |
|  |  |  | median (Q1-Q3) | 0.61 (0.24-0.92) | 1.00 (0.96-1.00) |  | 0.91 (0.68-1.00) | 1.00 (1.00-1.00) |  |
|  | **EQ-5D-5L VAS Score** |  | mean (SD) | 56.7 (21.6) | 91.4 (7.1) | **<.001** | 64.3 (20.7) | 91.9 (7.4) | **<.001** |
|  |  |  | median (Q1-Q3) | 50 (40-75) | 90 (90-97) |  | 65 (50-83) | 94 (90-97) |  |
|  | **Barthel Index Score** |  | mean (SD) | 64.7 (29.6) | 100.0 (0.0) | **<.001** | 82.9 (22.0) | 100.0 (0.0) | **<.001** |
|  |  |  | median (Q1-Q3) | 65 (40-95) | 100 (100-100) |  | 93 (78-100) | 100 (100-100) |  |

Stroke subjects, both with and without OD, divided into 2 subgroups based upon their MNA-SF score, with n=44 for score 0-11 (malnourished or at risk of malnutrition) and n=36 for score 12-14 (normal nourished) compared to their matched HR subjects

† 5 subjects had serum folate concentration above the upper limit of detection. In these cases, the upper limit of detection value was taken; 7 subjects had C-reactive protein concentration below the lower limit of detection. In these cases, values were replaced by half of the lower limit of detection.

Missing values (e.g. due to lack of volume) ranged between 0 and a maximum of 10 cases per parameter within all 160 subjects that were included in this subgroup analysis.

All *p* value were derived from a Wilcoxon signed rank test.

bold *p* values are <0.05.

Abbreviations: AA, Arachidonic acid; ALA, alpha-linolenic acid; DHA, docosahexaenoic acid; EPA, eicosapentaenoic acid; EQ-5D-5L, EuroQol 5-dimensions 5-level; LA, linoleic acid; MNA-SF, Mini Nutritional Assessment – Short Form; MUFA, monounsaturated fatty acids; n3, omega-3; n6, omega-6; PUFA, polyunsaturated fatty acids; Q1, Quartile 1; Q3, Quartile 3; SD, standard deviation; SFA, saturated fatty acids; VAS, visual analogue scale.
